# *Streptococcus suis* exports WapA polymorphic toxins to compete with tonsil microbiota for an optimal colonization

**DOI:** 10.1080/20002297.2025.2598988

**Published:** 2025-12-12

**Authors:** Xinming Pan, Jianan Liu, Ningyuan Zhong, Ruhui Fan, Zhen Zhang, Caiying Li, Huizhen Wu, Zongfu Wu, Qiankun Bai, Jiale Ma

**Affiliations:** a MOE Joint International Research Laboratory of Animal Health and Food Safety, College of Veterinary Medicine, Nanjing Agricultural University, Nanjing, China; b Key Lab of Animal Bacteriology, Ministry of Agriculture, Nanjing, China; c WOAH Reference Lab for Swine Streptococcosis, Bacterial Pathogenesis Research Group, Nanjing, China

**Keywords:** *Streptococcus suis*, WapA, polymorphic toxins, tonsil microbiota, sec pathway

## Abstract

**Background:**

*Streptococcus suis* is a zoonotic pathogen, and its colonization of the host tonsil is believed to be a vital source causing infection, while its mechanism competing for a stable tonsil niche is unknown. Rearrangement hotspot (Rhs) proteins are characterized to facilitate interbacterial competition by their polymorphic C-terminal toxins (CTs) in diverse bacteria, while their distant homologues emerged in *S. suis*, referred to as wall-associated protein A (WapA), has not been identified.

**Methods:**

Bioinformatics, western blot and interbacterial competition analyses were performed to identify Rhs/WapA toxins and their roles during *S. suis* infection.

**Results:**

The 350 kDa WapA-CT1, linked with a SecF-like protein and a SrtB sortase, was verified to manipulate the tonsil microbiota for *S. suis* optimal colonization. The unfolded WapA-CT1 was translocated across the cell membrane via the canonical Sec pathway. Afterward, autocleavage generated four fragments: the N-terminal NCWB fragment, two middle Rhs domains (Rhs1&2) that may fold as a *β*-barrel structure, and a C-terminal PreT-CT toxin domain. SrtB interacts with the NCWB region, and plays vital roles for the interbacterial antagonism mediated by the toxic CT1.

**Conclusion:**

This discovery underscores the diversity of mechanisms by which pathogens delivering Rhs/WapA polymorphic toxins, and their roles in competing with the host microbiota.

## Introduction

*Streptococcus suis*, an important pathogen in pigs, can cause severe conditions, including arthritis, sepsis and meningitis [1]. This zoonotic pathogen also poses a severe risk to human health, primarily causing meningitis and septic shock [2,3]. *S. suis* predominantly infect the host by colonizing the respiratory tract, with a strong presence in the tonsils and nasal passages [4]. Pig tonsils are inhabited by diverse microbiota and bacterial pathogens [5]. To achieve the most optimized survival in this densely colonized environment, *S. suis* deploys cell-surface proteins via the general secretory pathway (Sec) or delivers toxic effectors via the type VII secretion system (T7SS) to defend against host microbiota antagonism and to interact with host cells [6,7].

YD-peptide repeat proteins, which are universally encoded in both Gram-negative and -positive bacteria, are considered a class of highly selected polymorphic toxins deployed by bacteria for intercellular competition [8–11] and host invasion [12,13]. In Gram-negative bacteria, these proteins, labeled rearrangement hotspot (Rhs) proteins, possess a specific architecture comprising of a clade-specific *N*-terminal domain, an Rhs domain composed of conserved tyrosine‒aspartate (YD) repeats, and a divergent C-terminal toxin section delimited by the conserved peptide motif DPxG-x18-DPxG [9,14–16]. Previous research has shown that Rhs effectors associated with the type VI secretion system (T6SS) can undergo cleavage into three fragments, leading to the release of their C-terminal toxins [15–17]. This autoprocessing is initiated by an aspartyl protease domain that is present in homologous Rhs proteins such as the RhsP of *Vibrio parahaemolyticus* [17], the Tse5 of *Pseudomonas aeruginosa* [18], the RhsA of *Pseudomonas protegens* [19], and the Rhs1 of *Photorhabdus laumondii* [16]. In Gram-positive bacteria, their distant homologues also harbor an Rhs domain with conserved YD repeats and a divergent C-terminal toxin but exhibit variations in their *N*-terminal structure, notably featuring a signal peptide and a potential cell wall anchoring domain. Hence, these proteins are classified as wall-associated protein A (WapA) [10]. The WapA proteins are believed to traverse the cell membrane via the general secretory pathway (Sec system) guided by their N-terminal signal peptides, but the mechanisms facilitating the passage of these proteins, which are over 300  kDa in size, through the cell wall have yet to be definitively identified.

The canonical Sec pathway is crucial for translocating the majority of exported proteins across the cytoplasmic membrane in bacteria [20,21]. This pathway involves the integral membrane components SecYEG, which forms a channel through which unfolded proteins can traverse the membrane. The SecA ATPase also plays a critical role in this process by recognizing the specific N-terminal Sec signal peptide of substrates and providing the necessary energy for protein export. Furthermore, another heterotrimeric membrane complex, SecDF-YajC, functions as an auxiliary components that enhances translocation by utilizing the proton motive force [22,23]. The core components of the Sec translocon are found in all bacteria, while not all Gram-positive bacteria have SecD and SecF. For instance, bacteria such as *Streptococcus suis* and *Lactococcus lactis* lack these components. In Gram-positive bacteria, numerous surface proteins are exported via the Sec pathway, and are subsequently covalently linked to the cell wall by sortases [24], which are membrane-associated cysteine transpeptidase enzymes that catalyze the cleavage of their cognate substrates at a C-terminal cell wall sorting signal [25]. Sortase homologs and their substrates have been categorized into four groups [24]; among these, SrtA, known as the housekeeping sortase, anchors LPxTG signal motif-containing proteins to the bacterial cell wall. Accessory sortases, such as SrtB [26,27], are crucial components found in the same gene operons that encode their substrate proteins [26–29]. SrtB recognizes a variety of C-terminal sorting signals within different substrates, thereby facilitating the cell surface display of various proteins, including polymorphic toxins.

In this study, we identified a type of Rhs/WapA protein that is larger than 300 kDa and are encoded by conserved *srtB-secF-like-wapA* loci. WapA-CTs are responsible for facilitating interbacterial antagonism in *S. suis*, an important zoonotic pathogen [30], and then significantly facilitates the bacterial colonization by manipulating the tonsil microbiota. Subsequent studies were performed to clarify how this large type of polymorphic toxins was translocated across the cell membrane and thick envelope to compete for a stable niche with the host microbiota.

## Materials and methods

### Ethics approval

All the animal experiments were conducted in strict accordance with the animal welfare standards of the Animal Research Committee Guidelines of Jiangsu Province (License Number: SYXK (SU) 2022-0009) and were approved by the Ethics Committee for Animal Experimentation of Nanjing Agricultural University (permit no. NJAU. No 20250105110). All procedures followed strict regulations outlined by the relevant animal welfare agency to minimize harm to the animals.

### Bacterial strains and growth conditions

The *S. suis* isolates K56-WJ and WUSS351, which were stored in the OIE Reference Laboratory for Swine Streptococcosis, were used in this study. All these strains were cultured in Todd-Hewitt broth (THB, Oxoid Cheshire, UK) or THB agar (THA) at 37 °C with 5% CO_2_. Antibiotics and chemicals were added as needed, with chloramphenicol (Sigma–Aldrich) at 5  μg/mL, spectinomycin (Sigma–Aldrich) at 100  μg/mL, and sucrose at 10% (wt/vol) concentrations. Moreover, the *Escherichia coli* strain TOP10 was grown on Luria-Bertani (LB; Oxoid) medium at 37 °C. The antibiotic concentrations for *E. coli* were adjusted to ampicillin (Sigma–Aldrich) at 100  μg/mL, kanamycin at 50  μg/mL, rifampicin at 100 µg/mL, and spectinomycin at 50  μg/mL. For more details on the bacterial strains and plasmids utilized in this research, please refer to Table S1.

### DNA manipulations and plasmids construction

The oligonucleotide primers used in this study were synthesized by Genewiz Corporation (Suzhou, China) and are listed in Table S2. DNA amplification, ligation, and electroporation procedures were carried out following established protocols, with reference to a previous method unless otherwise specified [31]. All necessary restriction and DNA-modifying enzymes were obtained from Thermo Fisher Scientific (Waltham, USA) and used as per the manufacturer's guidelines. Deletion mutants were generated through the natural transformation method, following the procedures detailed in previous research conducted by our laboratory [32]. Initially, the forward and reverse homologous sequences of the target gene were combined with the spc or cat genes through overlap PCR. The resulting DNA products are subsequently combined with a specific peptide (a crucial element of the signal factor ComX for competence regulation) and bacterial cells before they undergo transformation. Positive clones were then selected on THB agar plates supplemented with either spectinomycin or chloramphenicol. Subsequent DNA sequencing was carried out by Sunny Biotechnology Corporation (Shanghai, China). The complemented strains were developed by performing isogenic replacement of the sequence with point mutations using the natural transformation method. To assess gene expression, the targeted ORF was inserted into the EcoRI and XhoI sites of the pBAD-HisA plasmid, which was subsequently introduced into *E. coli* Top10 for the expression of the target gene.

### Growth curves for bacterial toxicity assay

The plasmid pBAD/HisA (Invitrogen) was utilized to introduce the specified genes for the expression of effectors and the co-expression of effector‒immunity pairs. To detect *E. coli* growth curves, TOP10 cells containing the recombinant plasmids mentioned above were cultured overnight and then subcultured to an initial OD_600_ value of 0.05 in LB medium at 37 °C with agitation for 2.5 h. The target genes were subsequently induced by 0.2% (w/v) L-arabinose, while the vector pBAD/HisA was utilized as a control. Cell growth was monitored by measuring the OD_600_ value every 2 h. The findings presented in this study are the average values plus standard deviations (depicted as error bars) from three independent experiments.

### Western blot assays identifying the subcellular localization of WapA1-CT1

**Figure 1.**
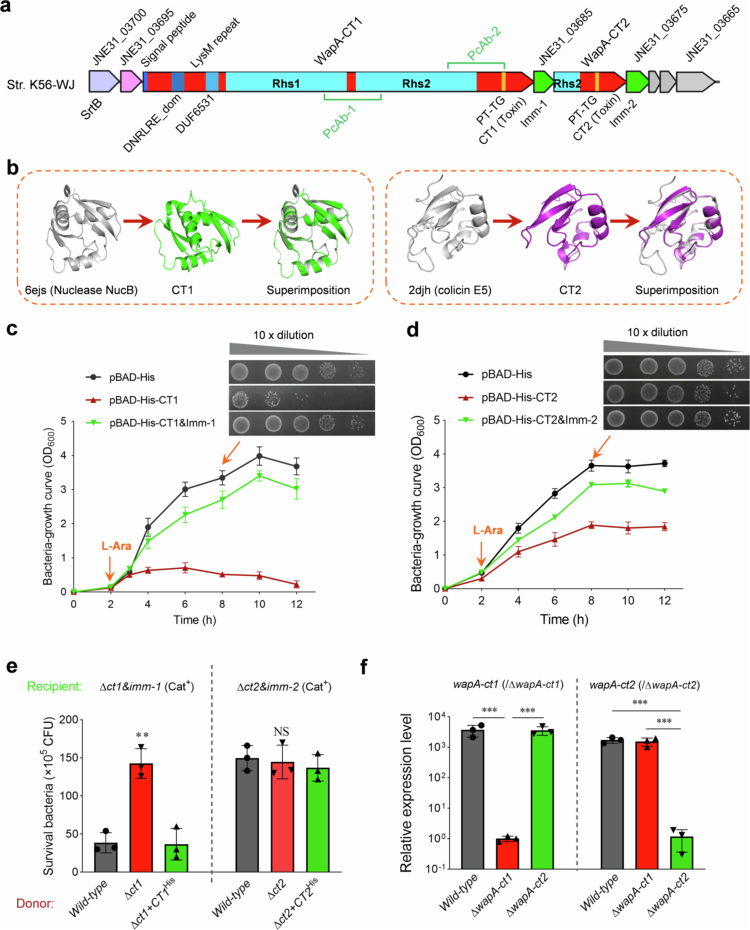
Identification of a Rhs/WapA family protein that plays an antibacterial role in *S. suis*. (a) Graphical depiction of a *rhs/wapA* gene locus in *S. suis* strain K56-WJ. The predicted novel toxin and immunity genes are indicated by red and green, respectively. (b) Ribbon depiction showing the superimposition of AlphaFold-predicted CT1 (colored green, pLDDT >85), CT2 (colored purple, pLDDT > 85) and the corresponding best-matching template (colored gray) was listed. (c, d) Growth curves of *E. coli* cells expressing the indicated toxins. The cultures were induced by L-arabinose at the indicated time (shown by the orange arrow). The bacterial load was monitored and the colony forming units was determined. (e) Interbacterial competition assays between the indicated *S. suis* donor and recipient strains. The donor and recipient were mixed at a ratio of 10:1 and incubated on solid medium for 22 h. Both the initial and final populations of each strain were enumerated by plating on selective medium. The full-length WapA-CT1 is more than 3,000 aa, causing the plasmid complementation that cannot be achieved. The *ct1-his-tag* sequence was inserted into the original coding position of the deletion mutant Δ*ct1* chromosome, forming the new gene *wapA-ct1-his-tag*. Therefore, the ∆*ct1* + CT1^His^ and ∆*ct2* + CT2^His^ are genomic complementary strains in situ, and a His tag was introduced to the C-termini of CT1 and CT2. (f) The relative transcriptional levels of *wapA-ct1* and *wapA-ct2* in the indicated strains detected by RT-qPCR assays. The data were normalized to the transcription level of the housekeeping gene *par C*. (c–f, Error bars indicate the mean ± standard deviation of three biological replicates. *p < 0.05; **p < 0.01; ***p < 0.001; NS, not significant.)

The bacterial culture of 500 mL was incubated to the logarithmic phase, then centrifuged at 12,000  rpm for 10 min. The subcellular proteins of each *S. suis* strains were then extracted as previously described [33]. The supernatant samples were further extracted using the classical TCA-acetone approach to acquire the secreted proteins. For fractionation of cell wall proteins, the bacterial pellets were resuspended in 2 mL of Buffer A (containing 30 mM Tris-HCl, 3 mM MgCl₂, 25% sucrose, and 125 U/mL lysostaphin, pH 7.4). The suspension was incubated at 37 °C for 60 min, followed by centrifugation at 12,000  rpm for 10 min at 4 °C. The resulting supernatant, containing the cell wall proteins, was then treated with 10% trichloroacetic acid (TCA) and kept on ice for 30 min to precipitate the proteins. The precipitates were subsequently washed twice with ice-cold acetone before the final collection of the cell wall proteins. Following the extraction, the samples were analyzed using conventional western blot assays with the mixed antibodies PcAb1&2 (Figure 1a) to identify the subcellular localization or secretion of WapA1-CT1. To serve as a loading control [34], an MRP-specific antibody was utilized for cytoplasmic and cell wall proteins, and an SLY-specific antibody was utilized for secreted proteins, revealing the specific cross-reactive bands. Additionally, an anti-DnaK antibody was employed as the internal reference for cytoplasmic proteins in order to evaluate the secreted protein samples.

### Co-immunoprecipitation assays

For strain K56-WJ, the total protein samples from the cell wall and cultural supernatant were extracted as described previously. The potentially cleaved fragments of WapA1-CT1 were then captured by immunoprecipitation (IP) using the mixed antibodies PcAb1&2. The product of IP was subsequently resolved on an SDS‒PAGE gel, and the protein bands were excised individually to determine the first 9 amino acids. This analysis was conducted using the automated Edman sequencing service provided by Beijing Bio-Tech Pack Technology Company Ltd.

### Interbacterial competition assay

For contact-dependent interbacterial antagonism [35], the donor and recipient strains were initially diluted in THB to a starting OD_600_ value of 0.6. The diluted strains were subsequently combined at a volume ratio of 10:1, after which the supernatant was removed through centrifugation at 7000  rpm. The resulting pellet was resuspended in 100  μL of phosphate-buffered saline (PBS). An 8 μL mixture of the resuspended cells was then spotted onto LB plates, followed by an incubation period of 22 h at 37 °C and 5% CO_2_. The spotted cells subsequently underwent serial dilution in PBS and were cultured on THB plates to enable the selection of recipient colonies using the appropriate antibiotic. The surviving recipient cell colony-forming units (CFU) were quantified on chloramphenicol plates. Notably, the experiment was repeated independently a minimum of three times to ensure the reliability and validity of the results.

### Oropharyngeal infection model of piglets and bacterial load analysis of tonsils

The oropharyngeal infection model detailed in previous studies [36,37] was employed with 20-day-old piglets. The bacterial strains used here were induced with rifampicin (RIF) resistance, allowing them to be easily distinguished from other bacteria within the oral microbiota. Prior to infection, oral samples were collected and assessed to ensure that no culturable RIF^+^ bacteria resided in the oral microbiota of the piglets. Briefly, the indicated bacterial solutions (3.5  mL) were sprayed in the oral cavities of healthy piglets (3 × 10^9^ CFU/pig) every 12 h for 4 days. The pharyngeal tonsils were subsequently collected from infected piglets (*n* = 3, each group), and homogenized in sterile PBS at a ratio of 1 g of tissue to 1  mL of PBS. The homogenates were then serially diluted 10-fold and plated on THB agar to count CFU.

### The levels of TNFα, IL-1β and IL-6 in the throat swabs

Throat swabs from the infected piglets were collected (*n* = 3, each group), and suspended within 100  μL of sterile PBS. The levels of cytokines in the supernatants were measured by ELISA kits for pig TNFα, IL-1β and IL-6 (BioLegend). The operation was performed according to the instructions, and the cytokine content in the supernatants was calculated after the standard curve.

### Tonsil microbiota analysis by 16S rRNA gene sequencing

The pharyngeal tonsils collected from infected piglets (*n* = 3, each group) were minced, weighed, and suspended at 1:9 (wt/vol) in sterile PBS. DNA was extracted directly from the tonsils for analysis using the cetyltrimethylammonium bromide method [38] and sent to Gene-Denovo Biotechnology Co., Ltd., for 16S rRNA gene sequencing. Sequence reads were grouped into operational taxonomic units (OTUs) at a sequence similarity level of ≥97% for a taxonomic assignment using Uparse (http://drive5.com/uparse/) [39], and species annotation was conducted through the Silva Database (https://www.arb-silva de/). The relative abundance of OTUs was represented as the mean ± SEM. To identify the differentially abundant microbiome species associated with the presence/absence of the *ct1* encoding sequence in *S. suis* strain K56-WJ, a MaAsLin2 analysis was performed to further correct for potential biological and technical confounders, including animal weight, library DNA concentration and sequencing read depth. *P* values were corrected for multiple testing using the Benjamini–Hochberg method and only species with a false discovery rate (FDR) <5% were considered significant.

### Bioinformatics analysis

The functions of the indicated proteins were predicted through the use of SWISS-MODEL (https://www.swissmodel.expasy.org/, probability > 50%) [40] or Phyre2 (confidence > 95%) [41]. Monomeric 3D protein structure predictions were carried out using AlphaFold on our local server, following the default parameters [42]. From a pool of multiple candidates, the final structural model was selected based on the highest Rama favored score and visualized using PyMOL (https://pymol.org/2/) for subsequent analysis. To construct the phylogenetic tree, MEGA 7.0 was employed with the neighbor-joining method (1000 bootstrap replicates; Poisson correction), and the tree was visualized using iTOL (https://itol.embl.de) [43].

### Protein purification and pull-down assay

To express the genes with either His or GST tags, they were first cloned into pGEX-4T-1 and pET28a vectors. The cultures were then grown to an OD600 of 0.6 in the presence of the appropriate antibiotics, followed by induction with 1 mM IPTG overnight at 20 °C. The cells were subsequently harvested by centrifugation at 6,000 × g for 15 min and resuspended in lysis buffer containing 20 mM Tris (pH 7.4), 500 mM NaCl, and 50 mM imidazole with a protease inhibitor. The resuspended cells were sonicated to facilitate target protein purification. After sonication, the cell lysates were centrifuged at 12,000 × g for 30 min at 4 °C. The resulting supernatants, which contained the recombinant 6xHis-tagged proteins or GST-tagged proteins, were purified using the HisTrap HP column and glutathione Sepharose according to the manufacturer's instructions from GE Healthcare. If the recombinant proteins were contained in the precipitates, they were purified after detergent solubilization and then used after protein renaturation. To quantify the protein content, Coomassie blue staining of the SDS‒PAGE gels and a Pierce BCA protein assay kit with dilution-free BSA protein standards from Thermo Fisher were used.

The direct interaction of the indicated protein pair was confirmed using the pull-down technique. For instance, 5  μg of purified NCWB1-C-GST protein was incubated in 600  μL of binding buffer (50  mM HEPES, pH 7.5, 250  mM NaCl) with 10  μg of rRhs2-His for 4 h, with rotation and the addition of 0.1  mM PMSF every hour. As a control, an empty GST-tag was utilized. The Anti-GST-Tag antibody (Abmart M20025) was diluted in 600  μL of binding buffer at a ratio of 1:500 and then incubated for 2 h on a rotator with prewashed protein A/G magnetic beads (MedChemExpress). The beads were subsequently washed three times. The protein mixtures were then incubated with beads coated with the Anti-GST-Tag Antibody for 3 h. Afterwards, the beads were washed six times with PBST. The bound proteins were eluted using 5 × SDS sample loading buffer, boiled for 10 min, and finally loaded onto a 12% SDS‒PAGE gel for western blot analysis. This methodology was replicated to analyze the interaction of other protein pairs.

### SPR analysis

Surface plasmon resonance experiments were conducted at 25 °C using a Biacore T200 instrument (GE Healthcare) in accordance with established protocols [44]. The CM5 sensory chips were employed to immobilize rNCWB1-*N* and rNCWB1-C individually at 600 response units (RUs). As a negative control, an uncoated "blank" channel was included in the setup. The analyte SrtB1 was diluted in running buffer (comprising 10  mM HEPES [pH 7.4], 150  mM NaCl, 3  mM EDTA, 0.05% Tween 20, and 5% dimethyl sulfoxide [DMSO]) and injected at various concentrations (2-fold dilutions from 4 to 256 nM) at a constant flow rate of 30 µL min^−1^. The obtained data were subsequently analyzed using the Biacore T200 evaluation software from GE Healthcare.

### Statistical analysis

The data were analyzed using Prism 8.0 (GraphPad) with an unpaired two-tailed Welch’s *t*-test for interbacterial competition and recipient survival assays. Significance was determined for *p*-values < 0.05 and denoted by asterisks (*p < 0.05, **p < 0.01, ***p < 0.001).

## Results

### Identification of a Rhs/WapA protein that mediates the interbacterial antagonism in *S. suis*

Polymorphic toxins, including Rhs/WapA proteins, are important weapons for bacteria to address intra- and interspecies conflicts [9,10]. Upon analysis of the *S. suis* strain K56-WJ genome, we identified two putative Rhs proteins with a typical architecture representing polymorphic toxins [45], JNE31_03690 and JNE31_03680 (Figure 1a), which we have denoted WapA-CT1 and WapA-CT2, respectively. WapA-CT1 is a 349-kDa protein comprising 3,158 amino acids with a DNRLRE domain, a LysM repeat domain, a DUF6531 domain, two Rhs domains, and a C-terminal extension. In contrast, WapA-CT2 consists of only a truncated Rhs domain and a distinct C-terminal extension. CT1 has significant structural similarity with the well-known Nuclease NucB (PDB: 6ejs), and CT2 has significant structural similarity with the DNase colicin E5 (Figure 1b). To confirm the toxic effects of these two C-terminal extensions (CT1 and CT2) of the Rhs/WapA proteins, we expressed them in the cytoplasm of *E. coli* LMG194 using the plasmid pBAD-His. Upon induction with L-arabinose, CT1 and CT2 significantly inhibited cell growth in *E. coli* (Figure 1c and 1d). However, co-expressing the adjacent Imm1 and Imm2 (potential cognate immunity proteins), respectively, partially alleviated the toxic effects on *E. coli* cells (Figure 1c and 1d). To validate the involvement of WapA-CT1 and WapA-CT2 in interbacterial antagonism, we generated recipient strains lacking any growth defects (Figure S1) by deleting the *ct1&imm1* pair or *ct2&imm2* pair, respectively. Unlike the wild-type strain, the ∆*ct1* strain displayed a significantly reduced capacity to kill the recipient strain ∆*ct1&imm1* on plate, and allelic complementation (plasmid complementation of a 9474 bp *wapA-CT1* was unavailable) of CT1 with a His tag fully restored this deficiency (Figure 1e left panel). However, compared with the wild-type strain, CT2 lacked the capacity to kill the recipient strain ∆*ct2&imm2* on plate; in comparison with the wild-type strain, the ∆*ct2* and ∆*ct2 + CT2*^*His*^ strains (Figure 1e right panel). RT-qPCR analyses revealed a slight change in the relative transcriptional levels of *wapA-ct2* compared to *wapA-ct1* in the wild-type strain (Figure 1f). This result suggests that CT2 does not have the ability to mediate competition, possibly because CT2 cannot be exported to target bacterial cells to exert its toxic effect. We analyzed the entire sequence of CT2 and found that it lacked the SignalP encoding sequence and that the Rhs-core domain was incomplete, which might have hindered its normal delivery. These “truncated duplications” have been reported as “Rhs orphans” in previous studies [46], suggesting that they likely result from rearrangements in *rhs/wapA* loci, leading to the acquisition of new effector‒immunity pairs while pushing the old pairs downstream.

### WapA-CT1 toxin manipulates the tonsil microbiota to facilitate niche competition of *S. suis* during the oropharyngeal infection

**Figure 2.**
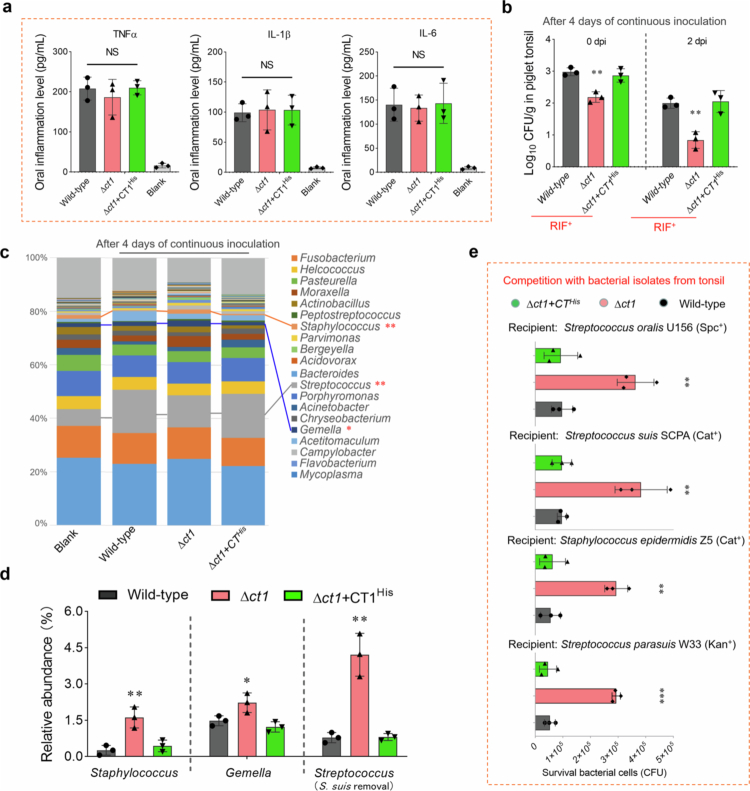
Identification of the roles of WapA-CT1 toxin during the oropharyngeal infection. The bacterial strains used here were induced with a rifampicin (RIF) resistance, allowing them to be easily distinguished from the other bacteria within the oral microbiota. Prior to infection, oral samples were collected and assessed to ensure that no culturable RIF^+^ bacteria resided in the oral microbiota of piglets. The indicated bacterial solutions (3.5  mL) were sprayed in the oral cavities of health piglets (3 × 10^9^ CFU/pig) every 12 h for 4 days. Throat swabs and pharyngeal tonsils were subsequently collected from infected piglets for following analyses. (a) The levels of cytokines in the normalized samples from throat swabs were measured by ELISA kits for pig TNFα, IL-1β and IL-6 (BioLegend). The throat swabs were collected within 100  μL of PBS on day 4 post-infection. MUC5AC levels were quantified using a MUC5AC/Mucin-5AC ELISA Kit (FineTest, China), with the sample concentrations adjusted through appropriate dilution for normalization. (b) THB plates with 100 µg/ml rifampicin were used to measure the bacterial loads for pharyngeal tonsils from the piglets infected with the indicated *S. suis* strains (*n* = 3, each group; RIF^+^, rifampicin resistant). The samples from uninfected piglets were confirmed to be free of rifampicin (100 µg/ml)-resistant bacteria. (c) The relative abundance of species in the communities of pharyngeal tonsils from the indicated groups (*n* = 3, each group). A MaAsLin2 analysis was performed to further correct for potential biological and technical confounders, with uninfected animals used as a reference. *P*-values were corrected for multiple testing using the Benjamini–Hochberg method, and only species with a false discovery rate (FDR) <5% were considered significant. The marked asterisks indicated that the relative abundances of genera in the ∆*ct1*-infected tonsils showed significant differences compared to the wild-type group, and were restored in the complementary ∆*ct1 + CT1*^*His*^ group. (d) The relative abundances of *Staphylococcus*, *Gemella* and *Streptococcus* (*S. suis* removal) in the communities of pharyngeal tonsils from the indicated groups. (e) Interbacterial competition with bacterial strains isolated from the tonsils of healthy pigs. These CT1-sensitive isolates were screened from the microbiome of pig tonsils. The assays were performed as outlined in the procedure of Figure 1e. (a–e, Statistical significance was calculated using an unpaired two-tailed Welch’s t-test. **p < 0.01; ***p < 0.001; NS, not significant.)

*S. suis* is an important zoonotic pathogen, and its colonization in host tonsil is believed to be a vital source of infection for humans and animals [38]. Here, we investigated whether WapA-CT1 toxin facilitate *S. suis* colonization in host oral and tonsils, based on its contribution to interbacterial antagonism. Given that the mouse model is not suitable for subsequent collection of tonsil samples, an oropharyngeal infection model detailed in previous studies [36,37] was employed with 20-day-old piglets. The bacterial solutions (3.5 mL) were sprayed in the oral cavities of healthy piglets (3 × 10^9^ CFU/pig) every 12 h for 4 days. Throat swabs were collected within 100  μL of PBS on day 4 post-infection, and the levels of internal reference protein by a MUC5AC/Mucin-5AC ELISA Kit (FineTest, China). Based on MUC5AC quantification, samples were normalized through appropriate dilution. These normalized samples were subsequently analyzed for cytokine levels using ELISA kits (BioLegend). Unexpectedly, no significant differences in the levels of TNFα, IL-1β and IL-6 among the wild-type, ∆*ct1* and ∆*ct1 + CT1*^*His*^ groups (Figure 2a). After 4 days of oropharyngeal inoculation of *S. suis* (RIF^+^, rifampicin resistant), bacterial load quantification in pharyngeal tonsils at 0 and 2 dpi (days postinfection) was measured through rifampicin resistance screening, which revealed significantly reduced bacterial counts of the ∆*ct1* mutant compared to the wild-type strain, while this deficiency was restored in the complementary strain ∆*ct1 + CT1*^*His*^ (Figure 2b). Prior to infection, oral samples were collected and assessed to ensure that no culturable RIF^+^ bacteria resided in the oral microbiota of the piglets. These results underscore the importance of the WapA-CT1 toxin in *S. suis* colonization within the host tonsil. Next, we compared the microbiota compositions of pharyngeal tonsils from the piglets infected with the indicated *S. suis* strains (*n* = 3, each group), using 16S rRNA gene sequencing. A MaAsLin2 analysis was performed to further correct for potential biological and technical confounders. The complete list of all the genera identified in the microbiomes of all the groups is summarized in Table S3. As shown in Figure 2c, compared with the microbial community of untreated tonsils, the most significantly increased genus was *Streptococcus* (from 6.25% to >15% relative abundance) in the *S. suis*-infected tonsils. The oral inoculation of *S. suis* was the main cause of the abnormal increase in *Streptococcus* abundance. However, compared with that of the ∆*ct1*-infected tonsils, the relative abundance of the *Streptococcus* genus after the removal of *S. suis* was significantly reduced from 4.23% to 0.78% and 0.81% in the wild-type and ∆*ct1 + CT1*^*His*^-infected tonsils (Figure 2d), respectively. In addition, the relative abundances of *Staphylococcus* and *Gemella* also significantly decreased in the groups with wild-type and ∆*ct1 + CT1*^*His*^ groups compared with the ∆*ct1*-infected group. These findings suggested that *Streptococcus, Staphylococcus* and *Gemella* bacteria may be targeted by CT1 toxin in host tonsils. To verify the role of CT1 in facilitating *S. suis* colonized within the host tonsil, several bacterial strains isolated from the tonsils of healthy pigs were employed as recipient strains for subsequent analysis. Unlike the wild-type strain, ∆*ct1* significantly impaired its capacity to kill the recipient strains on the plate, and its complementary strain ∆*ct1 + CT1*^*His*^ fully restored this deficiency (Figure 2e), suggesting that CT1 facilitates *S. suis* to obtain a survival advantage by mediating interbacterial antagonism to manipulate the tonsil microbiota.

### The Sec-dependent transport, cell wall anchoring and cleavage of Rhs/WapA protein

**Figure 3.**
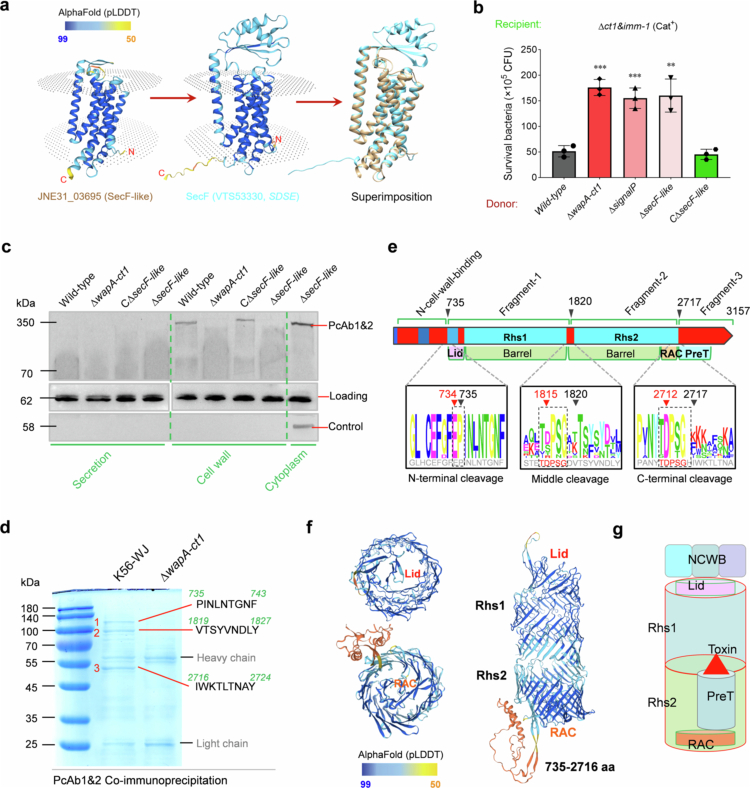
The subcellular localization analysis and autocleavage of Rhs/WapA protein. (a) Ribbon depiction showing the superimposition of the adjacent upstream JNE31_03695 and SecF homologue from *Streptococcus dysgalactiae*. The structures were predicted using AlphaFold with pLDDT confidence levels >90%. (b) The effects of the Sec secretion pathway on WapA-CT1 toxicity. C∆*secF-like* is a complementary strain of the deletion mutant ∆*secF-like*. The assays were performed as outlined in the procedure of Figure 1e. The error bars indicate the mean ± standard deviation of three biological replicates, ***p < 0.001. (c) Subcellular localization analysis of the WapA-CT1 effector in the indicated strains. Proteins were detected using the mixed antibodies PcAb1&2 prepared in this study against two fragments of WapA-CT1 (Figure 1a). An MRP-specific antibody was used as a loading control for cytoplasmic and cell wall proteins, and an SLY-specific antibody was utilized for secreted proteins [48]. Anti-DnaK antibody was used as a cytoplasmic sample control. (d) SDS–PAGE was used to analyze the cell wall and secretory protein samples purified by mixed antibodies PcAb1&2 mediating co-immunoprecipitation assay, and the excised protein bands were identified by automated Edman sequencing. (e) Schematic representation of the WapA-CT1 protein highlighting the different domains and the sequence conservation of N-, C-terminal and middle cleavage sites (logos, based on 10 homologues, including SUN07796, WNF86540, MBF0778746, CQR25028, ELT9178288, MDT2660328, MCJ1996141, QIW55055, QCE38859 and CYV11915). The sequences corresponding to the WapA-CT1 protein of *S. suis* strain K56-WJ are shown in gray below the logos. The black arrows indicate the cleavage sites, and the red arrows indicate the catalytic amino acid residues implicated in cleavage. (f) AlphaFold-predicted structures of Fragment 1&2 (735-2716 aa, containing the Rhs1 and Rhs2 domains). (g) Schematic model of the complex architecture of cleaved four fragments, NCWB, Rhs1, Rhs2 and PreT-CT1, based on our data and several previous studies [15–17]. NCWB is the N-terminal cell wall binding region, and RAC is the conserved Rhs repeat-associated core (RAC) domain located at the C-terminal end of Fragment 2.

The delivery mechanism is crucial for the toxins to kill adjacent bacteria and manipulate the host microbiota in the colonizing niche. The delivery mechanism of toxins to WapA-CT1, similar to homologues from *B. subtilis* [10,47], contains a canonical secretion signal sequence and is likely anchored to the peptidoglycan wall by the LysM repeat domain (Figure 1a), suggesting its secretion through the general secretory pathway (Sec system). The highly coincident superimposition between the AlphaFold-predicted structures of the adjacent upstream JNE31_03695 and SecF homologue from *Streptococcus dysgalactiae* (Figure 3a) reveals a potentially high degree of functional similarity between these two proteins. JNE31_03695 lacks the periplasmic domain characteristic of canonical SecF proteins and thus was redesigned as a SecF-like protein in this study. Given that the genes encoding core components of the Sec system are essential for bacterial survival, we consequently constructed individual deletion mutants of *signal-seq* encoding sequences (*N*-terminal 95 bp) or *secF-like*, as well as their complementary strains, to evaluate their impact on interbacterial competition. These deletion mutants did not present any growth defects (Figure S2), suggesting that their absence did not cause significant secondary effects on membrane integrity or the essential roles of Sec activity. Deletion of the SignalP-encoding sequence of *wapA-ct1*, or the *secF-like* gene, compromised the ability of WapA-CT1 to kill the recipient strain ∆*ct1&imm1* (Figure 3b), and the complement strain C∆*secF-like* restored the deficiency to a level similar to that of the wild-type strain, thereby suggesting the secretion of this putative effector via the Sec pathway. Given that the cell wall anchoring property is possibly mediated by the LysM repeat domain, we extracted supernatant and cell wall protein samples from the indicated strains to assess the presence or absence of WapA-CT1 using western blot analysis. As shown in Figure 3c, WapA-CT1 was undetectable in the cell wall and supernatant extractions from the ∆*wapA-ct1* and ∆*secF-like* mutant strains but was present in the cell wall samples of the wild-type and C∆*secF-like* strains, suggesting that WapA-CT1 translocated across the cell membrane and anchored to the peripeptidoglycan layer or reserving within the periplasm temporarily. Unlike the well-known Rhs effectors of the T6SS, WapA-CT1 and its cleaved small fragments were not detected in any of the supernatant samples for several proposed reasons: (1) potential low secretion below the detection threshold of western blot; (2) non-secretory wall anchoring protein in Gram-positive bacteria; (3) potential requirement of an alternative delivery pathway for crossing the cell wall layer; (4) degradation of cleaved small fragments.

Previous studies have shown that Rhs effectors of T6SS can undergo autocleavage into three fragments, enabling the release of their C-terminal toxins [15–17]. This type of autocleavage is triggered by an aspartyl protease domain, which is conserved in Rhs/YD-repeat domain. Unlike the reported Rhs proteins, WapA-CT1 possesses two distinct Rhs domains. Although WapA-CT1 is present mainly in the cell wall samples in full-length form prior to potential autocleavage (Figure 3c), we still speculated that its cleaved fragments may be released into the culture supernatant at very low levels below the detection limit of our secretion sample analysis. Therefore, co-immunoprecipitation assays were carried out using mixed antibodies PcAb1&2 to selectively enrich and purify fragments associated with WapA-CT1 from culture supernatant samples. The >3000 aa full-length WapA protein could not be made for the preparation of antiserum, and the immunized antigen fragments for the preparation of PcAb1&2 are shown in Figure 1a. The results revealed distinct bands on the SDS–PAGE gel for the wild-type and Δ*wapA-ct1* strains, labeled as fragment 1, 2 and 3 (Figure 3d), which were subjected to N-terminal amino acid sequencing analysis. The results determined that WapA-CT1 undergoes cleavage after glutamic acid at residue 735, aspartic acid at 1819, and histidine at 2716, leading to the generation of four fragments (Figure 3e): NCWB (~82 kDa, cell wall anchoring, non-secretory component), Rhs1 domain (~118 kDa, fragment 1, first 9 aa seq. PINLNTGNF), the Rhs2 domain (~100 kDa, fragment 2, first 9 aa seq. VTSYVNDLY), and the CT1 toxin domain (~50 kDa, fragment 3, first 9 aa seq. IWKTLTNAY). All three cleavage sites are located at the conserved signature regions of Rhs proteins, which is consistent with prior reports [15–17], and this cleavage event is likely to facilitate the liberation of the C-terminal toxin, suggesting its functional significance. Unlike the previously reported Rhs proteins, WapA-CT1 contains two Rhs domains, each of which encodes a conserved cleavage site “TDPSG” located at its C-terminus, leading to the generation of four fragments. The first cleavage site exhibits sequence identities with other known Rhs cleavages [15–17], all of which are situated at the N-terminus of a DUF6531 domain, while the first fragment NCWB is unique to WapA-CT1.

The size of full-length WapA-CT1 is too large to obtain a convincing structural prediction using the reported tools. However, the AlphaFold prediction of Fragment 1–2 clearly modeled the Rhs1-2 domains as a column of *β*-sheets (Figure 3f). Analogous to the RhsP of *Vibrio parahaemolyticus* [17], the N-terminal DUF6531 domain of Fragment 1 forms a Lid-like component to close the Rhs column, while the C-terminal end of Fragment 2 is completely sealed by a conserved Rhs repeat-associated core (RAC) domain (TIGRFAM: TIGR03696), which forms the bottom of *β*-barrel (Figure 3e and 3f). The Fragment 3 in both cleaved and uncleaved WapA-CT1 was predicted to be entirely encapsulated within the inner space enclosed by the *β*-barrel, Lid and RAC (Figure 3g), aligning with findings from previous studies [16,17], which plays a crucial role in maintaining the stability of the CT1 toxin during its transit. The *N*-terminal region (containing a PT-TG motif) of Fragment 3 was designated the PreT domain, which was inferred to facilitate the encapsulation of the C-terminal toxin domain by extensive contact with the inner surface of the *β*-barrel based on a recent study [17].

### Rhs/WapA proteins harbor diverse C-terminal toxins for interbacterial antagonism in *S. suis*

**Figure 4.**
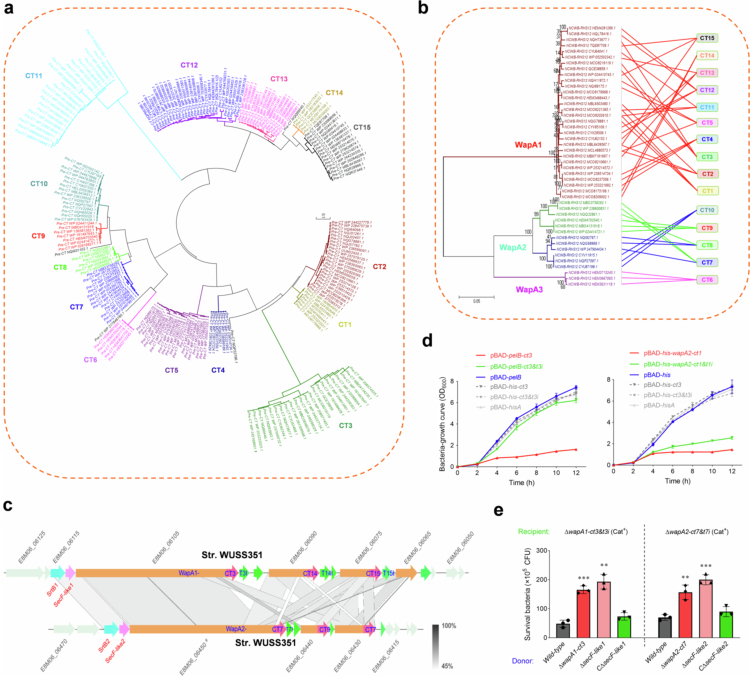
Identifying more WapA-CTs proteins across the *S. suis*. (a) Phylogenetic analysis of the C-terminal variable regions of Rhs/WapA family proteins widely encoded by *S. suis* isolates. A neighbor-joining tree (bootstrap *n* = 1000; Poisson correction) was constructed based on a ClustalW alignment of the amino acid sequences from the C-terminal toxins (CTs) of Rhs/WapA proteins using the MEGA software version 7.0. The deep clades are highlighted with distinct colors. (b) The interconnection diagram between CT types and the phylogenetic tree of N-terminal regions of Rhs/WapA proteins widely encoded in *S. suis* isolates. The detailed steps were performed as described in Figure 6a legend. (c) Graphical depiction of *wapA1-cts* and *wapA2-cts* loci encoded in *S. suis* strain WUSS351. The sequence identity between these two loci was showed in gray gradient following the corresponding ruler. (d) Growth curves of *E. coli* cells expressing the indicated toxins. Since CT3 and CT7 may exert effects only in the periplasmic space, their encoding sequences were then introduced into pBAD-*pelB* vector, incorporating a PelB signal peptide to direct its secretion into the periplasm [37]. The cultures were induced by L-arabinose at the indicated time (shown by the orange arrow). (e) Interbacterial competition assays between the indicated *S. suis* donor and recipient strains. The assays were performed as outlined in the procedure of Figure 1d. The error bars indicate the mean ± standard deviation of three biological replicates. **p < 0.01; ***p < 0.001.

A phylogenetic analysis was conducted on more than 200 Rhs/WapA family proteins from *S. suis* through MEGA 7.0 software. The C-terminal fusion sequences, potentially representing toxins, clustered into 15 distinct clades (Figure 4a), while the WapA sequences, consisting of N-terminal NCWB-Rhs1-Rhs2, were grouped into 3 distinct clusters (Figure 4b). Despite the diversity in the combinations of C-terminal fusion with WapA, different groups of WapA exhibited significant variations in CT toxins (Figure 4b). For instance, WapA1 harbored only CT1-5 and CT11-15 toxins, WapA2 harbored CT7-10 toxins, and the smallest branch, WapA3, harbored only one toxin, CT6. The *S. suis* strain WUSS351 was found to encode two distinct classes of Rhs/WapA family proteins. As illustrated in Figure 4c, the *wapA1* locus encoded a full-length WapA1-CT3/T3I toxin/immunity pair, along with two truncated duplications featuring the CT14/T14I and CT15/T15I pairs, respectively. Conversely, the *wapA2* locus encodes a full-length WapA2-CT7/T7I toxin/immunity pair and two truncated duplications with the CT9/T9I and CT7/T7I pairs, respectively. Previous analyses conducted in strain K56-WJ revealed that the downstream truncated gene *wapA1-ct2* cannot be delivered to mediate interbacterial competition (Figure 1e) and thus is nonfunctional. Consequently, only the WapA1-CT3/T3I and WapA2-CT7/T7I pairs were selected for further investigation. Cytoplasmic expression of *ct3* or *ct7* using the pBAD-His vector did not result in any growth inhibition in Top10 *E. coli* cells, suggesting that they may exert effects in the periplasmic space. A PelB signal peptide-encoding sequence was then introduced into the vector to direct the proteins into the periplasm [49]. Upon induction with L-arabinose, PelB-CT3 and PelB-CT7 significantly inhibited cell growth in *E. coli* Top10 (Figure 4d). Unlike the wild-type strain WUSS351, ∆*wapA1-ct3* displayed a significantly reduced capacity to kill the recipient strain ∆ *wapA1-ct3&t3i* on plates in competitive survival analyses (Figure 4e). Although the toxicity of the paired immune protein could not be neutralized, CT7 still showed significant efficacy in *E. coli* cytotoxicity and interbacterial competition (Figure 4d and 4e), indicating that the two distinct groups of Rhs/WapA family proteins could be effectively delivered without interference in the same strain.

### The cognate protein SrtB contribute to the delivery of the Rhs/WapA effector

**Figure 5.**
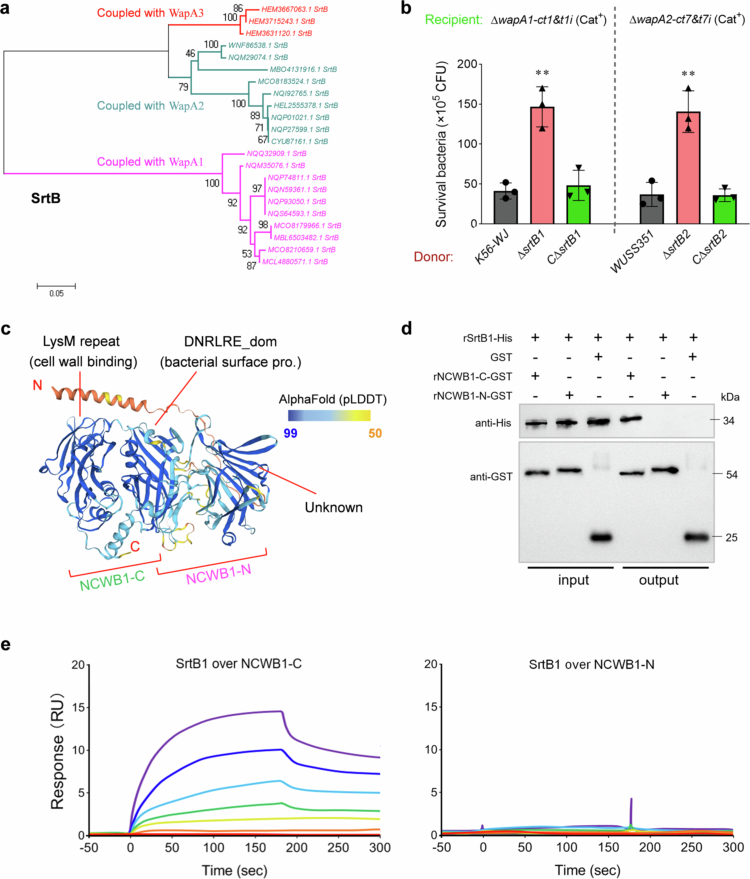
The roles of SrtB proteins on WapA-CTs export. (a) Phylogenetic analysis of the SrtB proteins coupled with different types of WapA proteins in *S. suis* isolates. The detailed steps were performed as described in Figure 4a legend. SrtB branches coupled with WapA1, 2 and 3 types are labeled purple, blue and red, respectively. (b) The effects of SrtB1 on WapA-CT1 toxicity, and SrtB2 on WapA-CT7 toxicity. C∆*srtB1* and C∆*srtB2* are the complementary strains of deletion mutants ∆*srtB1* and ∆*srtB2*, respectively. The assays were performed as outlined in the procedure of Figure 1e. The error bars indicate the mean ± standard deviation of three biological replicates. Statistical significance was calculated using an unpaired two-tailed Welch’s *t*-test, ***p < 0.001. (c) AlphaFold-predicted structures of the NCWB fragment (1-734 aa, containing the LysM repeat and DNRLRE domain). The model is shown as a ribbon representation and colored according to the AlphaFold pLDDT confidence level. (d) A pull-down assay detected the interaction between SrtB1 and NCWB1. Purified rNCWB1-*N* or rNCWB1-C-GST was incubated with rSrtB1-His, and the following steps were performed as described in the Figure 5d legend. (e) SPR sensorgram for SrtB1 analyte (2-fold dilutions; 4–256 nM) binding to immobilized NCWB1-C or -N ligand. The SPR responses are indicated in resonance units (RU).

In strain WUSS351, the *wapA1* and *wapA2* loci encode divergent upstream *secF-like* and *srtB* (Class B sortase, an optional protein of the Sec system) genes (Figure 4c). The deletion of *secF-like1* or *secF-like2* in strain WUSS351 hindered the killing of the indicated recipient strain mediated by WapA1-CT3 or WapA2-CT7 (Figure 4e), respectively, whereas the reintroduction of the corresponding *secF-like* gene reinstated this deficiency to a level similar to that of the wild-type strain. Indeed, phylogenetic analyses demonstrated that distinct groups of Rhs/WapA family proteins are coupled with specific SecF-like proteins (Figure S3), which are then designated cognate SecF-like1, 2 and 3. Similar phylogenetic branching was also observed for the SrtB proteins (Figure 5a), including SrtB1, 2 and 3. In strain K56-WJ, the depletion of SrtB1 has been shown to hinder the killing of the recipient strain ∆*wapA1-ct1&t1i* mediated by WapA1-CT1 (Figure 5b left panel), and the complementary strain C∆*srtB1* could restore the deficiency to a level similar to that of the wild-type strain. Similarly, in strain WUSS351, the depletion of SrtB2 impedes the toxicity of WapA2-CT7 (Figure 5b, right panel). SrtB is characterized as a membrane anchoring protein (Figure S4) belonging to the sortase superfamily, which consists of cysteine transpeptidases that are responsible for assembling surface proteins and pili on the bacterial cell envelope [26,50]. Genes coding for SrtB are often located within the same gene operons as their substrate proteins [26–29] and recognize diverse C-terminal cell wall sorting signals across various periplasmic substrates, a process crucial for cell surface display. In *S. suis*, WapA-CT1 is transported to the narrow space between the cell membrane and the cell wall. A protein with a length of more than 3100 amino acids may have significantly reduced autocleavage efficiency without the assistance of SrtB1. It is unable to anchor the cell wall and release CT1 and can only remain in this narrow space. Therefore, western blot analysis of the cell wall protein fraction (which might have contained WapA-CT1 that remained in this narrow space) failed to detect changes in WapA-CT1 levels resulting from *srtB1* deletion. Otherwise, the WapA-CT1 and its cleaved small fragments was not detected in any of the supernatant samples using the current technical methods employed by this study (Figure 3c), resulting in the failure of identifying whether the *srtB1* deletion inhibited the WapA-CT1 shifting from the cell wall to the culture supernatant. It is speculated based on several previous studies [15–17] that only a very small amount of CT1 toxin (the other fragments remaining on the cell wall) was released, and it might have exceeded the detection limit of the western blot test.

#### The localization of WapA-CT1 in Δ*srtB1* strain need to be further identified

In this study, NCWB was predicted as the *N*-terminal cell wall binding sequence. The AlphaFold-predicted structure of NCWB1 consists of three well-partitioned but similar domains (Figure 5c), including a C-terminal LysM repeat domain for cell wall binding, a middle DNRLRE_dom bacterial surface domain, and an unknown *N*-terminal domain. Pull-down analysis verified the interaction of SrtB1 with NCWB1-C (LysM repeat and DNRLRE domains) but not with NCWB1-*N* (Figure 5d). SPR analysis showed that the injection of SrtB1 across recombinant NCWB1-C generated signals in a dose-dependent manner (Figure 5e, left), whereas no such signals were observed with the NCWB1-*N*-immobilized chip (Figure 5e, right). SrtB needs to recognize a specific C-terminal sorting signal within its substrate and then catalyzes the production of a cell wall anchoring motif [26–29]. SrtB1 was shown to interact with the NCWB1 region before or after the cleavage of WapA1-CT1, and this interaction may facilitate the Rhs1&2-barrel to traverse the peptidoglycan layer and deliver the encapsulated C-terminal toxin. However, the current data are still insufficient to confirm that SrtB catalyzes NCWB to produce a cell wall anchoring motif, and the underlying mechanism needs to be further studied.

## Discussion

**Figure 6.**
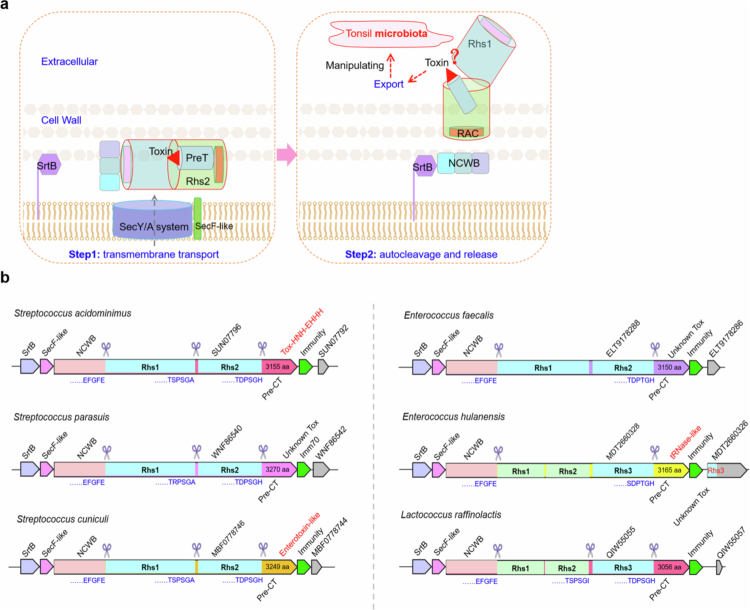
The delivery mechanism of WapA-CTs and its prevalence in Gram-positive bacteria. (a) Model depicting the delivery process of WapA-CTs. The delivery mechanism was described as below: Sec-dependent pathway across the cell membrane, SrtB interacting with NCWB, autocleavage into four fragments (NCWB, DUF6531-Rhs1, Rhs2 and PreT-CT), and Rhs1&2-barrel traversing the peptidoglycan layer for the delivery of encapsulated CT toxin. (b) Typical SrtB-SecF-like-WapA-CT modules in various Gram-positive bacteria. The potential cleavage sites are labeled with blue, the variable C-terminal extensions are labeled with different colors, and the putative immunity proteins are labeled with green arrows. The direction of the arrows indicates the direction of transcription.

In this work, we present a potential mechanism during the secretion of a Rhs/WapA polymorphic toxin that is linked with the canonical Sec translocon and potential autocleavage in *S. suis,* which significantly facilitates bacterial colonization in pharyngeal tonsils by manipulating the host microbiota. WapA-CT1 consists of four primary fragments: an N-terminal NCWB fragment containing a Sec-signal motif, a DNRLRE domain, and a LysM repeat domain responsible for cell wall anchoring; two middle Rhs fragments (Rhs1&2) folding into a *β*-barrel structure sealed by Lid/RAC plugs on either end; and a C-terminal PreT-CT toxin domain. Additionally, WapA-CT1 is coupled with a cognate SecF-like protein and a cognate SrtB sortase, which are encoded by two adjacent upstream genes. While the mode of translocation of WapA-CTs into recipient cells remains enigmatic, we propose a model (Figure 6a) in which (1) the unfolded WapA-CT proteins are translocated across the cell membrane utilizing the N-terminal Sec-signal motif and the cognate SecF-like protein through a Sec-dependent pathway, (2) the autocleavage of WapA-CTs into four fragments (NCWB, DUF6531-Rhs1, Rhs2 and PreT-CT), and (3) the interaction of SrtB with the NCWB before or after autocleavage may facilitate the Rhs1&2-barrel to traverse the peptidoglycan layer and deliver the encapsulated C-terminal toxins. Importantly, the *srtB-secF-like-wapA* loci are widespread in various Gram-positive bacterial species, particularly *Streptococcus*, *Enterococcus* and *Lactococcus* (Figure 6b), suggesting that analogous mechanisms may be broadly deployed by numerous bacteria to compete for an advantageous in complex ecological niches. A tonsil microbiota analysis based on 16S rRNA sequencing data revealed that WapA-CT1 facilitated *S. suis* to obtain a survival advantage by mediating interbacterial antagonism to manipulate the tonsillar microbiota. However, only *E. coli* and four strains (*S. oralis* U156, *S. suis* SCPA, *Staphylococcus epidermidis* Z5 and *S. parasuis* W33) isolated from pig tonsils were used as the target bacteria to assess toxin activity in this study. It is not yet known whether these inhibitory effects are relevant to the native tonsillar microbiota, where interspecies interactions could differ considerably.

Rhs proteins are sizable antibacterial toxins characterized by variable C-terminal domains that facilitate contact-dependent growth inhibition [11,51], and prior studies have reported a link between Rhs proteins and several secretion pathways, such as the T6SS and T7SS [9]. In Gram-negative bacteria, Rhs proteins are fused with several conserved PAAR domains in their *N*-termini, which bind to the *β*-needle tip of the VgrG transporter protein; thus, these proteins are recruited as T6SS effectors [11,15,16]. In contrast, most Rhs/WapA homologues in Gram-positive bacteria lack PAAR domains and are typically tagged with an *N*-terminal Sec-signal motif. The sizes of these proteins from Gram-positive bacteria are significantly larger (>300 kDa) compared to the Rhs effectors (~180 kDa) of the T6SS. However, a recent study identified a 188-kDa Rhs homologue, LrhA, in some *L. monocytogenes* isolates [52], which is encoded downstream of an EssC variant. Unlike typical Gram-positive Rhs proteins, LrhA does not have an *N*-terminal fusion of the Sec-signal and PAAR domains, while harbors variable C-terminal toxins in different strains. Furthermore, LrhA is located near genes encoding small WXG100-like proteins known for their roles as chaperones or targeting agents for T7-secreted toxins [48,53]. Although LrhA has not yet been experimentally confirmed, LrhA is predicted to be a potential T7SS effector. However, the Rhs/WapA-CT proteins lack the apparent T7SS helical targeting domain at the N-terminus, as do the three coupled small helical proteins that are typically present [52], suggesting that their secretion does not seem to be delivered via the T7SS transmembrane channel.

Rhs/WapA proteins from *B. subtilis* have been shown to deliver a variety of tRNase toxins that kill neighboring target bacterial cells [10,47] and appear to be exported with the guidance of their *N*-terminal Sec-signal. The Sec translocon, which is vital for protein export, consists of two principal components: the ATPase motor protein SecA and the protein-conducting channel SecYEG. Additionally, there is a non-essential membrane-bound complex SecDFYajC enhances Sec export efficiency [22,54]. Unexpectedly, SecDF is absent in *S. suis*. However, Rhs/WapA is coupled with a cognate SecF-like protein encoded by an adjacent upstream gene, which may form a membrane-bound complex with SecY to specifically stimulates the translocation of the coupled Rhs/WapA protein. Furthermore, the cognate sortase SrtB, which is encoded by an upstream gene, is indispensable for the export of Rhs/WapA. In addition to cleaving and anchoring the NCWB fragment to the cell wall, SrtB may also activate or enhance the cleavage efficiency of Rhs/WapA by interacting with the LysM repeat domain of the NCWB fragment, thus triggering the specific release of the C-terminal toxin. WapA-CT1 (~350 kDa) is much larger than other cell wall anchoring proteins. Without the assistance of SrtB1, WapA-CT1 cannot anchor to the cell wall, and it may also be unable to cross the cell wall layer and capsule to display its functional domain on the bacterial surface after crossing the cell membrane via the Sec pathway. Based on several previous studies of Rhs polymorphic toxins [15–17], we speculated that only a very small amount of CT1 toxin was released and that other fragments remained attached to the cell wall. These findings may explain why the deletion of *srtB1* does not alter the subcellular localization of WapA-CT1. Otherwise, a few *S. suis* strains, such as WUSS351, encode two *srtB-wapA* gene clusters, suggesting potential functional redundancy between SrtB1 and SrtB2. Different from the housekeeping sortase SrtA, which widely anchors LPxTG signal motif-containing proteins to the bacterial cell wall, the accessory sortase SrtB are crucial components found in the same gene operons that encode their cognate substrate proteins [26–29]. Given the relatively low sequence identity between the WapA1 and WapA2, SrtB1 and SrtB2 may not have functional redundancy.

Rhs/WapA proteins are proposed to translocate across the cell membrane in an unfolded form via the canonical Sec secretion pathway and then assume their 3D structure in the extracellular environment [10]. However, most full-length Rhs/WapA proteins are greater than 300 kDa in *S. suis*, thus an autocleavage may be required for the homologues to traverse the Gram-positive bacterial envelope. Autocleavage triggered by an aspartyl protease domain within the conserved YD-repeat region has been confirmed to cleave Rhs effectors of the T6SS into three fragments [15–17], which then form a complex with the *β*-needle tip of the VgrG transporter protein to be delivered for interbacterial competition. The autoclaved fragments, referred to as intramolecular chaperones, the auto-cleaved fragments facilitate the secretion of toxins, as they are encompassed entirely within the primary protein sequences [15,55]. Autocleavage is a common autoprocessing approach utilized by diverse proteases. For example, alpha-lytic protease and subtilisin use auto-cleaved peptides to guide the stability and folding of the protease domain [56,57]. Similarly, other proteases such as human carboxypeptidase Y and procathepsin L, are initially produced in an inactive precursor state to prevent unwanted toxicity; they are triggered to become active in response to specific environmental signals by cleaving off the inhibitory peptide [58,59].

## Conclusions

In summary, this study has delineated an example of translocating a large substrate in Gram-positive bacteria across the cell membrane and envelope through the canonical Sec secretion pathway and potential autocleavage. These findings underscore the diversity of mechanisms delivering Rhs polymorphic toxins, and their roles in bacterial pathogens competing for an optimal colonization with the host microbiota. Moving forward, it would be intriguing to uncover additional instances that employ a similar mechanism to ensure the effective delivery of toxic effectors.

## Supplementary Material

Supplementary material Supplementary tables and figures.

## Data Availability

Whole-genome sequences of *S. suis* strains K56-WJ and WUSS351 used in this study are available under the GenBank accession numbers NZ_JAEUXD000000000.1 and CP039462.1, respectively. Data that support the findings of this study are available in Figshare (http://doi.org/10.6084/m9.figshare.28794008).
