# Supplementary material for: Streptococcus suis exports WapA polymorphic toxins to compete with tonsil microbiota for an optimal colonization
Source: J Oral Microbiol. 2025 Dec 12;17(1):2598988. doi: 10.1080/20002297.2025.2598988 (PMC12704120; doi:10.1080/20002297.2025.2598988)
Supplement: Supplementary material — Supplementary tables and figures. [file ZJOM_A_2598988_SM5907.docx]

**Supplementary Figures**


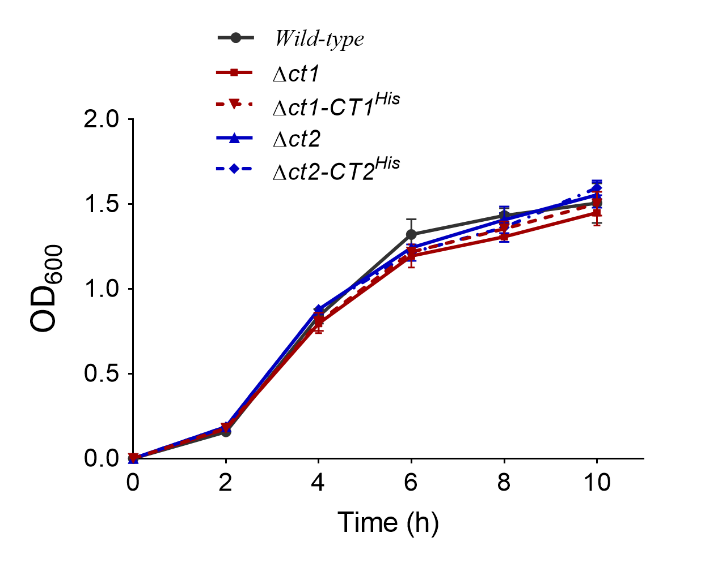


**Fig.** **S1** The growth curve of K56-WJ and its derived mutant strains (Δ*ct1*, Δ*ct1*+CT1^His^, Δ*ct2*, Δ*ct2*+CT2^His^). Error bars indicate the mean±standard deviation of three biological replicates.


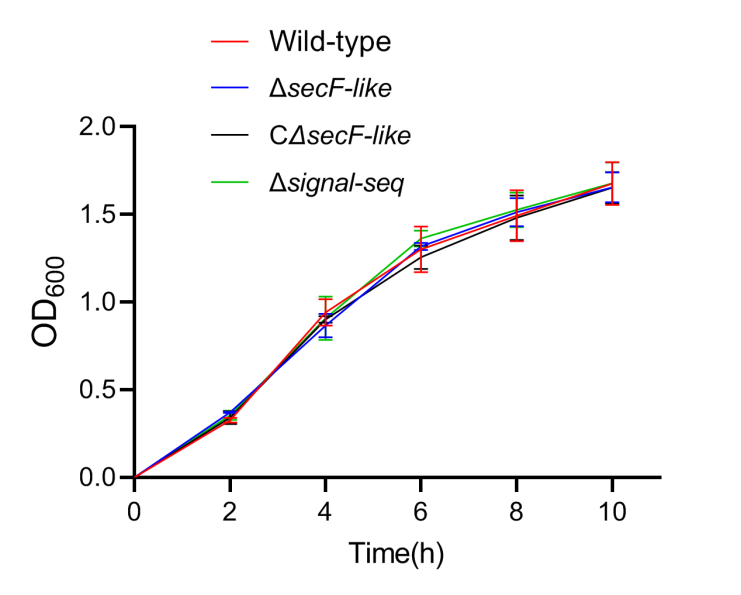


**Fig.** **S2** The growth curve of K56-WJ and its derived mutant strains (Δ*secF-like*, CΔ*secF-like*, Δ*signal-seq*). Error bars indicate the mean±standard deviation of three biological replicates.


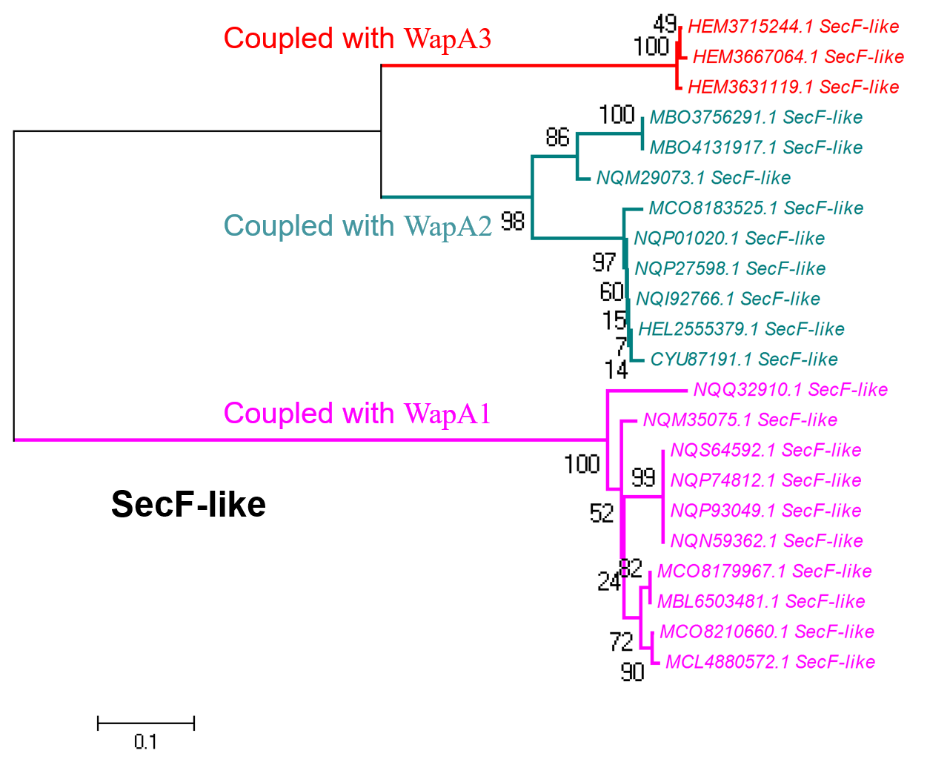


**Fig. S3** Phylogenetic analysis of the SecF-like proteins coupled with different types of WapA proteins in *S. suis* isolates. A neighbor-joining tree (bootstrap n = 1000; Poisson correction) was constructed based on a ClustalW alignment of the amino acid sequences using the MEGA software version 7.0. The deep clades were highlighted with distinct colors.


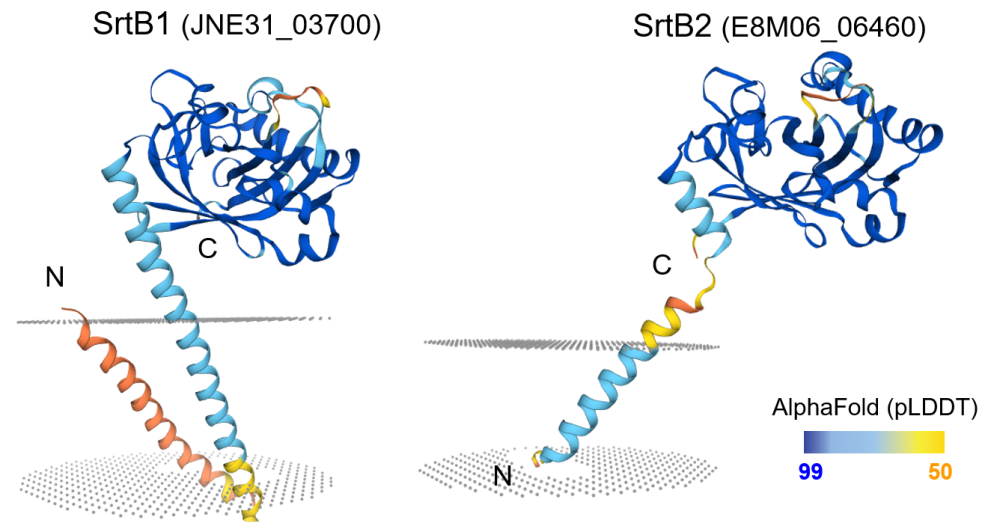


**Fig. S4** AlphaFold-predicted structures of SrtB1 and SrtB2. Model is shown as a ribbon representation and colored according to AlphaFold pLDDT confidence level.

**Table S1** A detailed list of bacterial strains and plasmids used in this study.

| Strains or plasmids | Strains or plasmids | Source or reference |
| --- | --- | --- |
| **Bacterial strains** |  |  |
| *K56-WJ* | *Streptococcus suis* strain | Our laboratory |
| *WUSS351* | *Streptococcus suis* strain | Our laboratory |
| E. coli Top10 | For cloning the recombinant plasmids | Purchased from Vazyme |
| E. coli BL21(DE3) | For cloning the recombinant plasmids | Purchased from Vazyme |
| E. coli DH5α | For cloning the recombinant plasmids | Purchased from Vazyme |
| E. coli BTH101 | For expressing the recombinant plasmids | Purchased from Euromedex, France |
| Δ*wapA-ct1* | Deletion mutant of *wapA-ct1* with *K56-WJ* background, Spc^+^ | This study |
| Δ*ct1* | Deletion mutant of *ct1* with *K56-WJ* background, Spc^+^ | This study |
| Δ*ct1&imm1* | Deletion mutant of *ct1 and imm1* with *K56-WJ* background, Cat^+^ | This study |
| Δ*ct1+CT1^His^* | Replenished of *CT1^His^* with Δ*ct1* mutant background, Spc^+^OptrA^+^ | This study |
| Δ*ct2* | Deletion mutant of *ct2* with *K56-WJ* background, Spc^+^ | This study |
| Δ*ct2&imm2* | Deletion mutant of *ct2* and *imm2* with *K56-WJ* background, Cat^+^ | This study |
| Δ*ct2+CT2^His^* | Replenished of *CT2^His^* with Δ*ct2* mutant background, Spc^+^OptrA^+^ | This study |
| Δ*yajC* | Deletion mutant of *yajC* with *K56-WJ* background, Spc^+^ | This study |
| CΔ*yajC* | Replenished of *yajC* using plasimd pSET-2S with Δ*yajC* mutant background, Spc^+^OptrA^+^ | This study |
| ΔsignalP | Deletion mutant of signalP encoding sequence of WapA-CT1 with *K56-WJ* background, Spc^+^ | This study |
| Δ*secF-like* | Deletion mutant of *ct2* with *K56-WJ* background, Spc^+^ | This study |
| CΔ*secF-like* | Replenished of *secF-like* using plasimd pSET-2S with Δ*secF-like* mutant background, Spc^+^OptrA^+^ | This study |
| Δ*wapA1-ct3* | Deletion mutant of *wapA1-ct3* with *WUSS351* background, Spc^+^ | This study |
| Δ*wapA1-ct3&t3i* | Deletion mutant of *wapA1-ct3* and *t3i* with *K56-WJ* background, Cat^+^ | This study |
| Δ*wapA2-ct7* | Deletion mutant of *wapA2-ct7* with *WUSS351* background, Spc^+^ | This study |
| Δ*wapA2-ct7&t7i* | Deletion mutant of *wapA2-ct7* and *t7i* with *K56-WJ* background, Cat^+^ | This study |
| Δ*srtB1^K56-WJ^* | Deletion mutant of *srtB1* with *K56-WJ* background, Spc^+^ | This study |
| CΔ*srtB1^K56-WJ^* | Replenished of *srtB1* using plasimd pSET-2S with Δ*srtB1* mutant background, Spc^+^OptrA^+^ | This study |
| Δ*srtB2^WUSS351^* | Deletion mutant of *srtB2* with *WUSS351* background, Spc^+^ | This study |
| CΔ*srtB2^WUSS351^* | Replenished of *srtB2* using plasimd pSET-2S with Δ*srtB2* mutant background, Spc^+^OptrA^+^ | This study |
| **Plasmid** |  |  |
| pET28a | His-tag expressing vector, Kan^R^ | Purchased from Vazyme |
| pBAD-HisA | His-tag expressing vector, Amp^R^ | Our laboratory |
| pSET-2S | *S. suis / E. coli* shuttle vector for complementation; Spc^R^ | This study |
| pGEX-4T-1 | GST-tag expressing vector, Amp^R^ | Our laboratory |
| PUT18C | B2H expression vector with *plac*, Amp^R^, C-terminal fusion to T18 fragment of CyaA | Purchased from Euromedex, France |
| PKT25 | B2H expression vector with *plac*, Kan^R^, C-terminal fusion to T25 fragment of CyaA | Purchased from Euromedex, France |
| pET28a-Rhs2 | pET28a carrying Rhs2 encoding fragment under the control of lactobiose promoter | This study |
| pET28a-NCWB | pET28a carrying NCWB encoding fragment under the control of lactobiose promoter | This study |
| pET28a-SrtB1 | pET28a carrying *srtB1* sequence under the control of lactobiose promoter | This study |
| pBAD::*CT1* | pBAD-HisA carrying *CT1* under the control of arabinose promoter | This study |
| pBAD::*CT1&Imm1* | pBAD-HisA carrying *CT1* and *Imm1* under the control of arabinose promoter | This study |
| pBAD::*CT2* | pBAD-HisA carrying *CT2* under the control of arabinose promoter | This study |
| pBAD::*CT2&Imm2* | pBAD-HisA carrying *CT2* and *Imm2* under the control of arabinose promoter | This study |
| pBAD::*CT7* | pBAD-HisA carrying *CT7* under the control of arabinose promoter | This study |
| pBAD::*CT7&**T7I* | pBAD-HisA carrying *CT7* and *T7I* under the control of arabinose promoter | This study |
| pBAD::*CT3* | pBAD-HisA carrying *CT3* under the control of arabinose promoter | This study |
| pBAD::*CT3&T3I* | pBAD-HisA carrying *CT3* and *T3I* under the control of arabinose promoter | This study |
| pGEX-4T-*NCWB1-C* | pGEX-4T carrying *NCWB1-C* encoding fragment under the control of lactobiose promoter | This study |
| pGEX-4T-*NCWB1-N* | pGEX-4T carrying *NCWB1-N* encoding fragment under the control of lactobiose promoter | This study |

Amp^R^, Ampicillin resistant; Kan^R^, Kanamycin resistant; Spc^R^ , spectinomycin resistant; Cat^R^, chloramphenicol resistant

**Table S2** Primers used in this study.

| Primers | Primers sequence (5’-3’) |
| --- | --- |
| **For deletion** |  |
| ∆*ct1*-1 | AAACCGCACATCGTCTGGTACTGA |
| ∆*ct1*-2 | ACATTATCCATTAAAAATCAAACAAATTTTCATCAAGCTCTAGTTCGGTGTGCCAGCCCAGTTGACTGCCTGAT |
| ∆*ct1*-3 | TGAACTCTATTCAGGAATTGTCAGATAGGCCTAATGACTGGCTTTTATAATCTTGGAGGTTCCGGCCTCCAATG |
| ∆*ct1*-4 | TTGAACTTGCGACCGCCTGTCCAT |
| ∆*ct1&imm1*-1 | TTCACGCAGAATGTCGTCCAG |
| ∆*ct1&imm1*-2 | CGTTATTAGTTATAGTTATTATAACATGTATTCACGAACACTAGTCTAACTCTGACTTTGACGGTA |
| ∆*ct1&imm1*-3 | GTAAATTTAACTATAAACTATTTAAATAACAGATTAAAAAAATTATAAGCAGGTGAAATTATCTCTCGT |
| ∆*ct1&imm1*-4 | TACCTTCTGATGATAGGCAGT |
| ∆*ct1*-1 | ATTGAAAGCATGGGCTATACGGTTAC |
| ∆*ct1*-2 | TAACATGTATTCACGAACACTAGTTTGACTACCTGGATAGGTGTCAG |
| ∆*ct1*-3 | TAACAGATTAAAAAAATTATAAGTGGCTCAAAAAGAGCGCATT |
| ∆*ct1*-4 | GTGGTATACCTCTTTGCATTAAAA |
| ∆*ct2&imm2*-1 | GCATTTCTGACGATTGATGTT |
| ∆*ct2&imm2*-2 | ACATTATCCATTAAAAATCAAACAAATTTTCATCAAGCTCTAGTTCGGTGTTTTGGCTGAGCGGGTCTGTT |
| ∆*ct2&imm2*-3 | TGAACTCTATTCAGGAATTGTCAGATAGGCCTAATGACTGGCTTTTATAATTGATAAGGAATCAATACCCTA |
| ∆*ct2&imm2*-4 | GTGTAGTTGGCTGGATTGTTCTGC |
| ΔwapA1-ct3-1 | TGTATTGTATTTTTTGTGC |
| ΔwapA1-ct3-2 | TAACATGTATTCACGAACACTAGAATTTTTCTTCTCTTTTT |
| ΔwapA1-ct3-3 | TAACAGATTAAAAAAATTATAAGAATGATTTGCAAAATTAC |
| ΔwapA1-ct3-4 | AACTTTTATACTTGTCATCTC |
| ΔwapA1-ct3&t3i-1 | TGTATTGTATTTTTTGTGC |
| ΔwapA1-ct3&t3i-2 | TCATCAAGCTCTAGTTCGGTGAATTTTTCTTCTCTTTTT |
| ΔwapA1-ct3&t3i-3 | CCTAATGACTGGCTTTTATAACAAATATCCTTGGGTGAGG |
| ΔwapA1-ct3&t3i-4 | GAGGTATACGGAGATTTGC |
| ΔwapA2-ct7-1 | ATGAAGTTTCTAAAAGTTAGC |
| ΔwapA2-ct7-2 | TAACATGTATTCACGAACACTAGTAATTTATGAAGTCGTTTC |
| ΔwapA2-ct7-3 | TAACAGATTAAAAAAATTATAAGTAATATTTAAGAGAGATTA |
| ΔwapA2-ct7-4 | CCTCCACCATAACTGCCTGAT |
| ΔwapA2-ct7&t7i-1 | ATGAAGTTTCTAAAAGTTAGC |
| ΔwapA2-ct7&t7i-2 | TCATCAAGCTCTAGTTCGGTGTAATTTATGAAGTCGTTTC |
| ΔwapA2-ct7&t7i-3 | CCTAATGACTGGCTTTTATAATGATTGTAAAAAGTATTTGAC |
| ΔwapA2-ct7&t7i-4 | AGCTAGAGCCCAACCGCGTTGTC |
| ∆*signalP-1* | ATGTATTGTATTTTTTGTGC |
| ∆*signalP-2* | TAACATGTATTCACGAACACTAGATATATCATCCTATTTATTT |
| ∆*signalP-3* | TAACAGATTAAAAAAATTATAAGAAATTGTGAAACCTCGCA |
| ∆*signalP-4* | GCGAATCTTTTCATTCCTAT |
| ∆*secF-like-1* | GGTCGATACGAACAGGA |
| ∆*secF-like-2* | TAACATGTATTCACGAACACTAGTTATTTACTTGAAAAAT |
| ∆*secF-like-3* | TAACAGATTAAAAAAATTATAAAAAAAATACAATACATCA |
| ∆*secF-like-4* | TACAGAATCTTCAGAAGTGA |
| ∆*yajC-1* | CTAACATCTAATTTGCTCCT |
| ∆*yajC-2* | TAACATGTATTCACGAACACTAGTGAATTGGCCAAAAAATTGA |
| ∆*yajC-3* | TAACAGATTAAAAAAATTATAATATAACTTTTCGTTATAACA |
| ∆*yajC-4* | ATCCTAACAGAAAAAACGAC |
| Spc-F | GAAATATAATGGTTCGGGGAA |
| Spc-R | TTATAATTTTTTTAATCTGTTA |
| Cm-F | CACCGAACTAGAGCTTGATG |
| Cm-R | TTATAAAAGCCAGTCATTAGG |
| SacB+Spc-F | GGATAATGCTGAAAACTCCTT |
| SacB+Spc-R | AATCTGATTACCAATTAGAATGAATAT |
| **For complementation strains** |  |
| CΔ*srtB1*-F | TATAAAATTTTTACAATTATGCTATGTATTGTATTTTTTGTGCATAT |
| CΔ*srtB1*-R | TTCTCTTTTCCATGGAGGATCCTTAGCTTCCTCCTTTTCTGAC |
| CΔ*srtB2*-F | TATAAAATTTTTACAATTATGCTTTTCTAAAAGTTAGCTTAGCGATG |
| CΔ*srtB2*-R | TTCTCTTTTCCATGGAGGATCCTCATGTATCTATCCTTTCTGTAAC |
| CΔ*secF-like*-F | TATAAAATTTTTACAATTATGCTATGGTTATTTTTAAAGAGAACAGA |
| CΔ*secF-like*-R | TTCTCTTTTCCATGGAGGATCCTTCAAGTAAATAATACACTGTCCC |
| CΔ*yajC*-F | TATAAAATTTTTACAATTATGCTGAAGGTTTATTTTTGCCACTT |
| CΔ*yajC*-R | TTCTCTTTTCCATGGAGGATCCTTATTCTTCAATTGCAGATTC |
| **For checking** |  |
| JC∆*ct* *1*-F | TGATAACCGTGTCTTCACTGC |
| JC∆*ct1*-R | GTGTGCTGATGATTGACATCG |
| JC∆*ct1&imm1*-F | TGTCCTCATCCCTTACACGA |
| JC∆*ct1&imm1*-R | TGAAGCCATACCAGAAGAGAC |
| JC∆*ct2*-F | TATACGGTTACCTACAATGGGA |
| JC∆*ct2*-R | TTTATAATCAGATTCGGGTGT |
| JC∆*ct2&imm2*-F | CATGGGGAGTCGTATGCAGGT |
| JC∆*ct2&imm2*-R | AAATAAGTGTAGAGGTTGTGC |
| JCΔwapA1-ct3-F | GGCAATGCGGTAAGGCAG |
| JCΔwapA1-ct3-R | AAAATCAACACCAGGATA |
| JCΔwapA1-ct3&t3i-F | GTTTCTACTTTATCTCCTACAG |
| JCΔwapA1-ct3&t3i-R | TACTCCATAATCTGTTTGTACT |
| JCΔwapA2-ct7-F | AGGAAATGTACACGCATG |
| JCΔwapA2-ct7-R | GCCATTTAAATAAGATTTT |
| JCΔwapA2-ct7&t7i-F | CGTTGCGACAGGGAATCCAAT |
| JCΔwapA2-ct7&t7i-R | CCCCATGGAGGAGTGGCTGAC |
| JC∆*secF-like-F* | GTAAAGGTTAAAGATGACCG |
| JC∆*secF-like-R* | ACTAGTTTTGAGGATCCTGT |
| JC∆*signalP-F* | ATGAAAAAGAGAAGAAAAAT |
| JC∆*signalP-R* | TTGAATTTCCTGCGCATAGA |
| JCΔ*yajC-F* | ATGATTGTAGAAAATCAATG |
| JCΔ*yajC-R* | TCAGTTGATGCAGCAGTTGT |
| JC-pBADhis-F | AGATTAGCGGATCCTACCTG |
| JC-pBAD-his-R | CACTTCTGAGTTCGGCATGG |
| Jc-pBAD-pelB-F | GCAGAAAAGTCCACATTGATTATTTGC |
| Jc-pBAD-pelB-R | TGCCTGGCAGTTCCCTACTCTCGCATG |
| **For plasmid** |  |
| pBAD-CT1-F | CGATGGGGATCCGAGCTCGAGGCCTATAACGCCGCTAAGAAAG |
| pBAD-CT1-R | AAACAGCCAAGCTTCGAATTCTTATTCCTCCGTAAATCTAAGCGTG |
| pBAD-CT1-Imm1-F | CGATGGGGATCCGAGCTCGAGGCCTATAACGCCGCTAAGAAAG |
| pBAD-CT1-Imm1-R | AAACAGCCAAGCTTCGAATTCCTGGTATTTCTTCAGTGCTTC |
| pBAD-CT2-F | TCTAGAGTCGACCTCGAGGTGGTCAATCCGCCGATCG |
| pBAD-CT2-R | AAACAGCCAAGCTTCGAATTCTCTAGGTCCTTTCCAGTTAGGATTATT |
| pBAD-CT2-Imm2-F | TCTAGAGTCGACCTCGAGGTGGTCAATCCGCCGATCG |
| pBAD-CT2-Imm2-R | AAACAGCCAAGCTTCGAATTCATGTGATAATACTTCAAAACA |
| pBAD-CT3-F | TCTAGAGTCGACCTCGAGGCCTATGTGCAGAACAACCCA |
| pBAD-CT3-R | AAACAGCCAAGCTTCGAATTCATGTAATTTTGCAAATCATTC |
| pBAD-CT3-T3I-F | TCTAGAGTCGACCTCGAGGCCTATGTGCAGAACAACCCA |
| pBAD-CT3-T3I-R | AAACAGCCAAGCTTCGAATTCTCACCCAAGGATATTT |
| pBAD-CT7-F | CGATGGGGATCCGAGCTCGAGGCTAAGAGCATGTTCCGTAAC |
| pBAD-CT7-R | AAACAGCCAAGCTTCGAATTCTAATCTCTCTTAAATATTACT |
| pBAD-CT7-T7I-F | CGATGGGGATCCGAGCTCGAGGCTAAGAGCATGTTCCGTAAC |
| pBAD-CT7-T7I-R | AAACAGCCAAGCTTCGAATTCAATACTTTTTACAATCACATTTTG |
| PUT18C::CT1-F | tctagaggatccccgggtaccTGGAAGACTTTGACAAATGCATAT |
| PUT18C::CT1-R | cttagttatatcgatgaattcTTCCTCCGTAAATCTAAGCGTGAC |
| PUT18C::Rhs1-F | tctagaggatccccgggtaccGAACGAACAGATGTATCTATCTCA |
| PUT18C::Rhs1-R | cttagttatatcgatgaattcGTCTCTGTCGAGAGACTACCAGTT |
| PUT18C::Rhs2-F | tctagaggatccccgggtaccCAAGCTATGTCAATGACCTCTACG |
| PUT18C::Rhs2-R | cttagttatatcgatgaattcGATATGCCCACTCGGGTCGATGTA |
| PUT18C::NCWB-F | tctagaggatccccgggtaccCCCCTGGTCTATGCGCAGGAA |
| PUT18C::NCWB-R | cttagttatatcgatgaattcAAATTCACAGTGTAAACCACG |
| pGEX-4T-*NCWB1-C*-F | ccgcgtggatccccggaatAAGGTCAACTATGACTTTAAGCAG |
| pGEX-4T-*NCWB1-C*-R | acgatgcggccgctcgagAAATTCACAGTGTAAACCACG |
| pGEX-4T-*NCWB1-N*-F | ccgcgtggatccccggaatCCCCTGGTCTATGCGCAGGAA |
| pGEX-4T-*NCWB1-N*-R | acgatgcggccgctcgagATACATACGACTACGACCGAT |
| pET28a-Rhs2-F | agcaaatgggtcgcggatcc CAAGCTATGTCAATGACCTCTACG |
| pET28a-Rhs2-R | gtggtggtggtggtgctcgag GATATGCCCACTCGGGTCGATGTA |
| pET28a-NCWB-F | agcaaatgggtcgcggatccCCCCTGGTCTATGCGCAGGAA |
| pET28a-NCWB-R | gtggtggtggtggtgctcgagAAATTCACAGTGTAAACCACG |
| pET28a-SrtB1-F | agcaaatgggtcgcggatccATGTATTGTATTTTTTGTGCATAT |
| pET28a-SrtB1-R | gtggtggtggtggtgctcgagGCTTCCTCCTTTTCTGAC |
| **For qPCR** |  |
| QP-CT1-F | GGTACTGAGATACGCTCACAAA |
| QP-CT1-R | GCCGGAACCTCCAAGATATAAG |
| QP-CT2-F | CATTATGTTGCAGTCGACGATAG |
| QP-CT2-R | CTAGGTCCTTTCCAGTTAGGATT |

**Table S3** The list of all genuses identified in the tonsil microbiota from the piglets infected with the indicated bacterial strains (n=3, each group).

| **Genus** | **Blank-1** | **Blank-2** | **Blank-3** | **Wild-type (WT)-1** | **Wild-type (WT)-2** | **Wild-type (WT)-3** | **Δ*ct1*-1** | **Δ*ct1*-2** | **Δ*ct1*-3** | ***P* value**  **(Δct1 vs WT)** | **Δ*ct1*+CT1^His^-1** | **Δ*ct1*+CT1^His^-2** | **Δ*ct1*+CT1^His^-3** | ***P* value**  **(Δ*ct1*+CT1^His^ vs WT)** |
| --- | --- | --- | --- | --- | --- | --- | --- | --- | --- | --- | --- | --- | --- | --- |
| Bacteroides | 23.86% | 25.21% | 22.56% | 23.00% | 22.16% | 23.84% | 25.31% | 23.83% | 24.79% | 0.1193 | 22.24% | 24.36% | 22.53% | 0.2521 |
| Fusobacterium | 11.63% | 11.93% | 10.23% | 11.58% | 13.66% | 10.68% | 11.92% | 12.55% | 11.29% | 0.1551 | 10.54% | 11.67% | 9.65% | 0.1083 |
| Streptococcus | 6.20% | 6.30% | 6.25% | 16.20% | 15.12% | 17.28% | 10.37% | 11.27% | 12.27% | 0.0089 | 16.47% | 14.26% | 17.12% | 0.1178 |
| Streptococcus (except  *S. suis*) | 3.47% | 4.03% | 2.91% | 0.78% | 0.99% | 0.57% | 4.23% | 5.18% | 3.31% | 0.0066 | 0.81% | 0.95% | 0.66% | 0.1698 |
| Helcococcus | 4.93% | 3.30% | 4.00% | 4.79% | 4.33% | 5.25% | 4.73% | 3.63% | 5.03% | 0.2252 | 4.37% | 5.02% | 4.26% | 0.1245 |
| Porphyromonas | 9.38% | 8.74% | 9.52% | 8.02% | 8.52% | 7.52% | 8.13% | 8.03% | 7.20% | 0.1767 | 8.81% | 8.71% | 7.94% | 0.0824 |
| Pasteurella | 5.99% | 4.26% | 6.72% | 4.04% | 4.56% | 3.52% | 4.02% | 4.31% | 3.73% | 0.1337 | 3.93% | 3.56% | 4.18% | 0.1113 |
| Acinetobacter | 2.56% | 2.01% | 3.11% | 1.18% | 0.99% | 1.32% | 1.64% | 1.42% | 1.86% | 0.0383 | 2.00% | 1.13% | 1.55% | 0.0485 |
| Moraxella | 3.09% | 3.56% | 2.62% | 2.20% | 2.51% | 1.89% | 2.03% | 2.68% | 2.38% | 0.1315 | 2.24% | 2.62% | 1.86% | 0.0983 |
| Chryseobacterium | 2.03% | 2.13% | 1.93% | 1.48% | 1.69% | 1.27% | 1.40% | 1.66% | 1.14% | 0.0949 | 1.91% | 1.28% | 1.58% | 0.0615 |
| Actinobacillus | 2.72% | 2.22% | 3.22% | 1.38% | 1.19% | 1.57% | 1.65% | 1.06% | 1.74% | 0.1154 | 1.22% | 1.53% | 1.81% | 0.1049 |
| Gemella | 1.38% | 1.21% | 1.55% | 1.48% | 1.68% | 1.27% | 2.24% | 2.72% | 1.81% | 0.0158 | 1.22% | 1.44% | 1.01% | 0.1854 |
| Peptostreptococcus | 1.01% | 0.49% | 0.02% | 0.36% | 0.44% | 0.28% | 0.54% | 0.61% | 0.49% | 0.0687 | 0.58% | 0.62% | 0.54% | 0.0698 |
| Acetitomaculum | 0.96% | 1.54% | 1.25% | 2.74% | 2.99% | 2.49% | 2.04% | 2.72% | 2.56% | 0.0889 | 2.02% | 2.53% | 1.51% | 0.0885 |
| Staphylococcus | 1.01% | 1.37% | 1.73% | 0.26% | 0.46% | 0.06% | 1.62% | 2.35% | 1.18% | 0.0078 | 0.44% | 0.68% | 0.20% | 0.1756 |
| Campylobacter | 0.88% | 0.96% | 0.92% | 1.16% | 1.02% | 1.28% | 1.46% | 1.31% | 1.11% | 0.0588 | 1.04% | 1.09% | 1.39% | 0.1054 |
| Parvimonas | 0.45% | 0.39% | 0.51% | 0.72% | 0.84% | 0.60% | 0.92% | 0.62% | 0.92% | 0.0987 | 0.74% | 0.82% | 0.68% | 0.0754 |
| Flavobacterium | 0.50% | 0.41% | 0.59% | 0.52% | 0.41% | 0.63% | 0.60% | 0.41% | 0.79% | 0.0587 | 0.46% | 0.55% | 0.37% | 0.0873 |
| Bergeyella | 0.32% | 0.39% | 0.25% | 0.28% | 0.25% | 0.31% | 0.38% | 0.28% | 0.28% | 0.1042 | 0.30% | 0.00% | 0.50% | 0.0875 |
| Mycoplasma | 0.27% | 0.31% | 0.23% | 0.14% | 0.11% | 0.17% | 0.14% | 0.19% | 0.09% | 0.1243 | 0.18% | 0.02% | 0.14% | 0.0911 |
| Acidovorax | 0.24% | 0.22% | 0.26% | 0.40% | 0.52% | 0.28% | 0.28% | 0.31% | 0.24% | 0.0697 | 0.26% | 0.31% | 0.21% | 0.0648 |
| Paracoccus | 0.24% | 0.18% | 0.30% | 0.32% | 0.42% | 0.25% | 0.26% | 0.51% | 0.31% | 0.0654 | 0.46% | 0.41% | 0.51% | 0.0531 |
| Proteocatella | 0.22% | 0.19% | 0.25% | 0.40% | 0.35% | 0.45% | 0.30% | 0.35% | 0.42% | 0.0521 | 0.10% | 0.13% | 0.07% | 0.0198 |
| Soonwooa | 0.22% | 0.27% | 0.17% | 0.28% | 0.18% | 0.38% | 0.26% | 0.21% | 0.31% | 0.1683 | 0.34% | 0.41% | 0.28% | 0.1658 |
| Veillonella | 0.06% | 0.09% | 0.03% | 0.08% | 0.02% | 0.05% | 0.02% | 0.04% | 0.00% | 0.0578 | 0.06% | 0.02% | 0.10% | 0.1036 |
| Trueperella | 0.18% | 0.25% | 0.11% | 0.04% | 0.00% | 0.14% | 0.52% | 0.55% | 0.39% | 0.0098 | 0.34% | 0.50% | 0.38% | 0.0086 |
| Leptotrichia | 0.17% | 0.11% | 0.23% | 0.16% | 0.11% | 0.21% | 0.46% | 0.51% | 0.41% | 0.0145 | 0.45% | 0.46% | 0.45% | 0.0123 |
| Thermomonas | 0.17% | 0.22% | 0.12% | 0.30% | 0.41% | 0.19% | 0.20% | 0.15% | 0.25% | 0.0324 | 0.10% | 0.14% | 0.06% | 0.0106 |
| Rhizorhapis | 0.14% | 0.09% | 0.19% | 0.22% | 0.19% | 0.25% | 0.16% | 0.19% | 0.21% | 0.0613 | 0.26% | 0.22% | 0.30% | 0.0648 |
| Luteimonas | 0.13% | 0.17% | 0.09% | 0.30% | 0.38% | 0.22% | 0.22% | 0.40% | 0.34% | 0.0785 | 0.14% | 0.16% | 0.12% | 0.0231 |
| Alloprevotella | 0.12% | 0.13% | 0.11% | 0.20% | 0.15% | 0.25% | 0.06% | 0.09% | 0.03% | 0.0185 | 0.01% | 0.05% | 0.04% | 0.0118 |
| Ralstonia | 0.11% | 0.07% | 0.15% | 0.40% | 0.50% | 0.30% | 0.04% | 0.00% | 0.08% | 0.0054 | 0.00% | 0.00% | 0.00% | 0.0058 |
| Psychrobacter | 0.10% | 0.15% | 0.05% | 0.10% | 0.15% | 0.05% | 0.10% | 0.08% | 0.12% | 0.1563 | 0.18% | 0.21% | 0.15% | 0.0689 |
| Filifactor | 0.09% | 0.11% | 0.07% | 0.10% | 0.08% | 0.12% | 0.14% | 0.18% | 0.10% | 0.1324 | 0.02% | 0.04% | 0.00% | 0.0187 |
| Rhodobacter | 0.08% | 0.11% | 0.05% | 0.06% | 0.10% | 0.02% | 0.08% | 0.05% | 0.03% | 0.0978 | 0.12% | 0.16% | 0.08% | 0.0261 |
| Brevundimonas | 0.08% | 0.04% | 0.12% | 0.04% | 0.00% | 0.08% | 0.08% | 0.13% | 0.03% | 0.1254 | 0.16% | 0.21% | 0.13% | 0.0304 |
| Prevotella | 0.08% | 0.10% | 0.06% | 0.10% | 0.15% | 0.05% | 0.00% | 0.00% | 0.00% | 0.0089 | 0.06% | 0.07% | 0.03% | 0.0398 |
| Filobacterium | 0.08% | 0.05% | 0.11% | 0.20% | 0.24% | 0.06% | 0.00% | 0.00% | 0.00% | 0.0051 | 0.10% | 0.07% | 0.13% | 0.0245 |
| Enhydrobacter | 0.08% | 0.03% | 0.13% | 0.06% | 0.02% | 0.10% | 0.10% | 0.13% | 0.07% | 0.0564 | 0.14% | 0.17% | 0.11% | 0.0824 |
| Actinomyces | 0.05% | 0.07% | 0.09% | 0.02% | 0.00% | 0.04% | 0.16% | 0.11% | 0.21% | 0.0189 | 0.08% | 0.11% | 0.05% | 0.0387 |
| Pseudoxanthomonas | 0.05% | 0.06% | 0.07% | 0.12% | 0.06% | 0.18% | 0.08% | 0.19% | 0.04% | 0.0865 | 0.04% | 0.07% | 0.11% | 0.1005 |
| Catonella | 0.05% | 0.09% | 0.01% | 0.00% | 0.00% | 0.00% | 0.00% | 0.00% | 0.00% | ﹣ | 0.20% | 0.15% | 0.25% | 0.0054 |
| Fretibacterium | 0.04% | 0.05% | 0.06% | 0.02% | 0.02% | 0.02% | 0.00% | 0.00% | 0.03% | 0.1826 | 0.00% | 0.00% | 0.00% | 0.0098 |
| Alcaligenes | 0.05% | 0.08% | 0.02% | 0.02% | 0.01% | 0.03% | 0.16% | 0.11% | 0.21% | 0.0098 | 0.00% | 0.00% | 0.00% | 0.0105 |
| Comamonas | 0.06% | 0.04% | 0.05% | 0.12% | 0.10% | 0.14% | 0.17% | 0.16% | 0.09% | 0.0885 | 0.04% | 0.01% | 0.07% | 0.0188 |
| Faecalibacterium | 0.05% | 0.09% | 0.01% | 0.12% | 0.15% | 0.09% | 0.06% | 0.10% | 0.02% | 0.0756 | 0.00% | 0.00% | 0.00% | 0.0086 |
| Stenotrophobacter | 0.07% | 0.05% | 0.03% | 0.04% | 0.03% | 0.05% | 0.05% | 0.04% | 0.04% | 0.0654 | 0.04% | 0.08% | 0.00% | 0.1068 |
| Pseudomonas | 0.05% | 0.07% | 0.03% | 0.02% | 0.04% | 0.00% | 0.01% | 0.07% | 0.03% | 0.0754 | 0.06% | 0.09% | 0.03% | 0.1036 |
| Peptoniphilus | 0.04% | 0.06% | 0.05% | 0.06% | 0.09% | 0.03% | 0.04% | 0.08% | 0.00% | 0.0873 | 0.08% | 0.12% | 0.04% | 0.1005 |
| Halomonas | 0.05% | 0.05% | 0.05% | 0.00% | 0.00% | 0.00% | 0.18% | 0.18% | 0.15% | 0.0023 | 0.18% | 0.15% | 0.21% | 0.0036 |
| Brochothrix | 0.03% | 0.05% | 0.07% | 0.00% | 0.00% | 0.00% | 0.00% | 0.00% | 0.00% | ﹣ | 0.00% | 0.00% | 0.00% | ﹣ |
| Globicatella | 0.05% | 0.08% | 0.02% | 0.04% | 0.07% | 0.01% | 0.06% | 0.09% | 0.03% | 0.0668 | 0.00% | 0.00% | 0.00% | 0.0087 |
| Capnocytophaga | 0.08% | 0.04% | 0.00% | 0.06% | 0.09% | 0.03% | 0.04% | 0.08% | 0.00% | 0.0912 | 0.06% | 0.04% | 0.08% | 0.0787 |
| Pelistega | 0.04% | 0.06% | 0.02% | 0.02% | 0.03% | 0.01% | 0.04% | 0.04% | 0.01% | 0.0854 | 0.00% | 0.00% | 0.00% | 0.0098 |
| Aeromonas | 0.01% | 0.07% | 0.04% | 0.00% | 0.00% | 0.00% | 0.13% | 0.17% | 0.05% | 0.0088 | 0.10% | 0.16% | 0.04% | 0.0087 |
| Tannerella | 0.04% | 0.04% | 0.04% | 0.04% | 0.01% | 0.07% | 0.04% | 0.02% | 0.09% | 0.0956 | 0.02% | 0.04% | 0.00% | 0.0848 |
| Alcanivorax | 0.04% | 0.07% | 0.01% | 0.00% | 0.00% | 0.00% | 0.18% | 0.21% | 0.18% | 0.0032 | 0.12% | 0.15% | 0.09% | 0.0053 |
| Proteus | 0.03% | 0.05% | 0.01% | 0.00% | 0.00% | 0.00% | 0.00% | 0.00% | 0.00% | ﹣ | 0.00% | 0.00% | 0.00% | ﹣ |
| Stenotrophomonas | 0.05% | 0.03% | 0.01% | 0.00% | 0.00% | 0.00% | 0.19% | 0.09% | 0.25% | 0.0018 | 0.07% | 0.16% | 0.10% | 0.0086 |
| Sphingobacterium | 0.03% | 0.02% | 0.04% | 0.02% | 0.03% | 0.01% | 0.04% | 0.00% | 0.04% | 0.1123 | 0.03% | 0.01% | 0.03% | 0.1148 |
| Niabella | 0.00% | 0.06% | 0.03% | 0.02% | 0.02% | 0.02% | 0.02% | 0.04% | 0.00% | 0.0921 | 0.08% | 0.02% | 0.04% | 0.0731 |
| Blastocatella | 0.03% | 0.03% | 0.03% | 0.04% | 0.01% | 0.07% | 0.06% | 0.10% | 0.02% | 0.0854 | 0.02% | 0.00% | 0.04% | 0.0698 |
| Mitsuaria | 0.03% | 0.05% | 0.01% | 0.04% | 0.05% | 0.03% | 0.02% | 0.07% | 0.04% | 0.1766 | 0.04% | 0.08% | 0.00% | 0.1658 |
| Alicycliphilus | 0.06% | 0.03% | 0.00% | 0.10% | 0.08% | 0.12% | 0.00% | 0.00% | 0.00% | 0.0068 | 0.00% | 0.00% | 0.00% | 0.0072 |
| Spongiibacter | 0.03% | 0.00% | 0.06% | 0.00% | 0.00% | 0.00% | 0.00% | 0.00% | 0.00% | ﹣ | 0.00% | 0.00% | 0.00% | ﹣ |
| Delftia | 0.01% | 0.03% | 0.05% | 0.02% | 0.01% | 0.03% | 0.01% | 0.03% | 0.03% | 0.0879 | 0.00% | 0.00% | 0.00% | 0.0089 |
| Fastidiosipila | 0.00% | 0.06% | 0.03% | 0.08% | 0.10% | 0.06% | 0.02% | 0.00% | 0.04% | 0.0358 | 0.00% | 0.00% | 0.00% | 0.0096 |
| Lysinibacillus | 0.03% | 0.02% | 0.01% | 0.04% | 0.02% | 0.06% | 0.06% | 0.03% | 0.05% | 0.1235 | 0.04% | 0.08% | 0.00% | 0.1475 |
| Brachymonas | 0.02% | 0.03% | 0.01% | 0.04% | 0.05% | 0.03% | 0.02% | 0.04% | 0.00% | 0.0984 | 0.02% | 0.04% | 0.00% | 0.0566 |
| Shinella | 0.03% | 0.01% | 0.02% | 0.04% | 0.03% | 0.05% | 0.04% | 0.05% | 0.03% | 0.1365 | 0.00% | 0.00% | 0.00% | 0.0088 |
| Diaphorobacter | 0.02% | 0.02% | 0.02% | 0.04% | 0.06% | 0.02% | 0.04% | 0.07% | 0.01% | 0.1456 | 0.00% | 0.00% | 0.00% | 0.0087 |
| Moheibacter | 0.01% | 0.02% | 0.03% | 0.00% | 0.00% | 0.00% | 0.00% | 0.00% | 0.00% | ﹣ | 0.06% | 0.09% | 0.03% | 0.0085 |
| Johnsonella | 0.02% | 0.02% | 0.02% | 0.06% | 0.05% | 0.07% | 0.02% | 0.00% | 0.08% | 0.213 | 0.00% | 0.00% | 0.00% | 0.0086 |
| Tetrasphaera | 0.02% | 0.03% | 0.01% | 0.00% | 0.00% | 0.00% | 0.06% | 0.03% | 0.09% | 0.0036 | 0.02% | 0.01% | 0.04% | 0.0099 |
| Hydrogenophaga | 0.03% | 0.02% | 0.01% | 0.06% | 0.08% | 0.04% | 0.05% | 0.04% | 0.06% | 0.1013 | 0.00% | 0.00% | 0.00% | 0.0097 |
| Arenimonas | 0.02% | 0.01% | 0.03% | 0.00% | 0.00% | 0.00% | 0.02% | 0.02% | 0.04% | 0.0095 | 0.06% | 0.07% | 0.04% | 0.0054 |
| Mesorhizobium | 0.03% | 0.01% | 0.02% | 0.02% | 0.04% | 0.00% | 0.05% | 0.05% | 0.01% | 0.2134 | 0.06% | 0.09% | 0.03% | 0.0562 |
| Ottowia | 0.02% | 0.03% | 0.01% | 0.00% | 0.00% | 0.00% | 0.00% | 0.00% | 0.00% | ﹣ | 0.00% | 0.00% | 0.00% | ﹣ |
| Eubacterium | 0.01% | 0.00% | 0.02% | 0.04% | 0.01% | 0.07% | 0.00% | 0.00% | 0.00% | 0.0098 | 0.02% | 0.01% | 0.03% | 0.0212 |
| Blastomonas | 0.01% | 0.02% | 0.00% | 0.00% | 0.00% | 0.00% | 0.00% | 0.00% | 0.00% | ﹣ | 0.02% | 0.00% | 0.04% | 0.0132 |
| Succiniclasticum | 0.01% | 0.01% | 0.01% | 0.04% | 0.04% | 0.04% | 0.03% | 0.00% | 0.05% | 0.1121 | 0.02% | 0.04% | 0.00% | 0.1884 |
| Granulicatella | 0.01% | 0.02% | 0.00% | 0.02% | 0.01% | 0.03% | 0.04% | 0.08% | 0.00% | 0.0787 | 0.00% | 0.00% | 0.00% | 0.0102 |
| Aquabacterium | 0.01% | 0.02% | 0.00% | 0.02% | 0.02% | 0.02% | 0.00% | 0.00% | 0.00% | 0.0098 | 0.02% | 0.00% | 0.04% | 0.1339 |
| Qipengyuania | 0.01% | 0.00% | 0.02% | 0.02% | 0.03% | 0.01% | 0.02% | 0.04% | 0.00% | 0.1568 | 0.02% | 0.04% | 0.00% | 0.1442 |
| Fusibacter | 0.01% | 0.00% | 0.02% | 0.00% | 0.00% | 0.00% | 0.00% | 0.00% | 0.00% | ﹣ | 0.00% | 0.00% | 0.00% | ﹣ |
| Lachnoanaerobaculum | 0.01% | 0.01% | 0.01% | 0.06% | 0.03% | 0.09% | 0.00% | 0.00% | 0.00% | 0.0096 | 0.00% | 0.00% | 0.00% | 0.0093 |
| Neisseria | 0.01% | 0.01% | 0.01% | 0.06% | 0.09% | 0.03% | 0.00% | 0.00% | 0.00% | 0.0085 | 0.00% | 0.00% | 0.00% | 0.0085 |
| Suttonella | 0.01% | 0.00% | 0.02% | 0.00% | 0.00% | 0.00% | 0.00% | 0.00% | 0.00% | ﹣ | 0.00% | 0.00% | 0.00% | ﹣ |
| Proteiniphilum | 0.01% | 0.01% | 0.01% | 0.00% | 0.00% | 0.00% | 0.00% | 0.00% | 0.00% | ﹣ | 0.00% | 0.00% | 0.00% | ﹣ |
| Klebsiella | 0.01% | 0.02% | 0.00% | 0.02% | 0.01% | 0.03% | 0.03% | 0.02% | 0.03% | 0.0956 | 0.02% | 0.03% | 0.01% | 0.0881 |
| Terrimonas | 0.01% | 0.01% | 0.01% | 0.00% | 0.00% | 0.00% | 0.00% | 0.00% | 0.00% | ﹣ | 0.00% | 0.00% | 0.00% | ﹣ |
| Roseivirga | 0.01% | 0.00% | 0.02% | 0.00% | 0.00% | 0.00% | 0.04% | 0.06% | 0.02% | 0.0086 | 0.04% | 0.07% | 0.01% | 0.0102 |
| Pyramidobacter | 0.01% | 0.01% | 0.01% | 0.04% | 0.06% | 0.02% | 0.00% | 0.00% | 0.00% | 0.0123 | 0.00% | 0.00% | 0.00% | 0.0153 |
| Finegoldia | 0.01% | 0.02% | 0.00% | 0.00% | 0.00% | 0.00% | 0.00% | 0.00% | 0.00% | ﹣ | 0.00% | 0.00% | 0.00% | ﹣ |
| Streptobacillus | 0.01% | 0.01% | 0.01% | 0.04% | 0.00% | 0.08% | 0.00% | 0.00% | 0.00% | 0.0316 | 0.00% | 0.00% | 0.00% | 0.0389 |
| Peptococcus | 0.01% | 0.00% | 0.02% | 0.04% | 0.08% | 0.00% | 0.00% | 0.00% | 0.00% | 0.0423 | 0.00% | 0.00% | 0.00% | 0.0412 |
| Peredibacter | 0.01% | 0.01% | 0.01% | 0.00% | 0.00% | 0.00% | 0.00% | 0.00% | 0.00% | ﹣ | 0.00% | 0.00% | 0.00% | ﹣ |
| Sphingorhabdus | 0.01% | 0.00% | 0.02% | 0.02% | 0.00% | 0.04% | 0.00% | 0.00% | 0.03% | 0.0986 | 0.02% | 0.00% | 0.04% | 0.0797 |
| Silanimonas | 0.01% | 0.02% | 0.00% | 0.02% | 0.04% | 0.00% | 0.04% | 0.01% | 0.04% | 0.1211 | 0.02% | 0.04% | 0.00% | 0.0864 |
| Kurthia | 0.00% | 0.00% | 0.00% | 0.00% | 0.00% | 0.00% | 0.00% | 0.00% | 0.00% | ﹣ | 0.02% | 0.03% | 0.01% | ﹣ |
| Lactococcus | 0.00% | 0.00% | 0.00% | 0.00% | 0.00% | 0.00% | 0.02% | 0.01% | 0.03% | ﹣ | 0.00% | 0.00% | 0.00% | ﹣ |
| Methylobacterium | 0.00% | 0.00% | 0.00% | 0.00% | 0.00% | 0.00% | 0.02% | 0.03% | 0.01% | ﹣ | 0.00% | 0.00% | 0.00% | ﹣ |
| Leuconostoc | 0.00% | 0.00% | 0.00% | 0.00% | 0.00% | 0.00% | 0.00% | 0.00% | 0.00% | ﹣ | 0.02% | 0.00% | 0.04% | ﹣ |
| Lysobacter | 0.00% | 0.00% | 0.00% | 0.00% | 0.00% | 0.00% | 0.00% | 0.00% | 0.00% | ﹣ | 0.02% | 0.01% | 0.03% | ﹣ |
| Leadbetterella | 0.00% | 0.00% | 0.00% | 0.02% | 0.00% | 0.04% | 0.00% | 0.00% | 0.00% | ﹣ | 0.00% | 0.00% | 0.00% | ﹣ |
| Porphyrobacter | 0.00% | 0.00% | 0.00% | 0.02% | 0.04% | 0.00% | 0.00% | 0.00% | 0.00% | ﹣ | 0.00% | 0.00% | 0.00% | ﹣ |
| Sphingopyxis | 0.00% | 0.00% | 0.00% | 0.02% | 0.03% | 0.01% | 0.00% | 0.00% | 0.00% | ﹣ | 0.00% | 0.00% | 0.00% | ﹣ |
| Paludibacterium* | 0.00% | 0.00% | 0.00% | 0.00% | 0.00% | 0.00% | 0.00% | 0.00% | 0.00% | ﹣ | 0.02% | 0.03% | 0.01% | ﹣ |
| Devosia | 0.00% | 0.00% | 0.00% | 0.00% | 0.00% | 0.00% | 0.00% | 0.00% | 0.00% | ﹣ | 0.02% | 0.00% | 0.04% | ﹣ |
| Runella | 0.00% | 0.00% | 0.00% | 0.00% | 0.00% | 0.00% | 0.00% | 0.00% | 0.00% | ﹣ | 0.02% | 0.02% | 0.02% | ﹣ |
| Cloacibacterium | 0.00% | 0.00% | 0.00% | 0.00% | 0.00% | 0.00% | 0.00% | 0.00% | 0.00% | ﹣ | 0.02% | 0.01% | 0.03% | ﹣ |
| Alteromonas | 0.00% | 0.00% | 0.00% | 0.00% | 0.00% | 0.00% | 0.02% | 0.02% | 0.02% | ﹣ | 0.00% | 0.00% | 0.00% | ﹣ |
| Haematospirillum | 0.00% | 0.00% | 0.00% | 0.00% | 0.00% | 0.00% | 0.00% | 0.00% | 0.00% | ﹣ | 0.02% | 0.00% | 0.04% | ﹣ |
| Nitrospina | 0.00% | 0.00% | 0.00% | 0.00% | 0.00% | 0.00% | 0.02% | 0.01% | 0.03% | ﹣ | 0.00% | 0.00% | 0.00% | ﹣ |
| Ferruginibacter | 0.00% | 0.00% | 0.00% | 0.00% | 0.00% | 0.00% | 0.00% | 0.00% | 0.00% | ﹣ | 0.02% | 0.01% | 0.03% | ﹣ |
| Deinococcus | 0.00% | 0.00% | 0.00% | 0.00% | 0.00% | 0.00% | 0.02% | 0.00% | 0.04% | ﹣ | 0.00% | 0.00% | 0.00% | ﹣ |
| Carnobacterium | 0.00% | 0.00% | 0.00% | 0.00% | 0.00% | 0.00% | 0.00% | 0.00% | 0.00% | ﹣ | 0.02% | 0.03% | 0.01% | ﹣ |
| Gulbenkiania | 0.00% | 0.00% | 0.00% | 0.00% | 0.00% | 0.00% | 0.00% | 0.00% | 0.00% | ﹣ | 0.02% | 0.02% | 0.02% | ﹣ |
| Dokdonella | 0.00% | 0.00% | 0.00% | 0.00% | 0.00% | 0.00% | 0.02% | 0.02% | 0.02% | ﹣ | 0.00% | 0.00% | 0.00% | ﹣ |
| Romboutsia | 0.00% | 0.00% | 0.00% | 0.02% | 0.00% | 0.04% | 0.00% | 0.00% | 0.00% | ﹣ | 0.00% | 0.00% | 0.00% | ﹣ |
| Cutibacterium | 0.00% | 0.00% | 0.00% | 0.00% | 0.00% | 0.00% | 0.02% | 0.03% | 0.01% | ﹣ | 0.00% | 0.00% | 0.00% | ﹣ |
| Achromobacter | 0.00% | 0.00% | 0.00% | 0.00% | 0.00% | 0.00% | 0.00% | 0.00% | 0.00% | ﹣ | 0.02% | 0.03% | 0.01% | ﹣ |
| Atopobium | 0.00% | 0.00% | 0.00% | 0.00% | 0.00% | 0.00% | 0.02% | 0.02% | 0.02% | ﹣ | 0.00% | 0.00% | 0.00% | ﹣ |
| Other | 13.19% | 14.76% | 15.93% | 12.17% | 10.36% | 12.98% | 9.23% | 6.76% | 10.55% | 0.0395 | 13.46% | 11.37% | 15.86% | 0.0321 |

Note：A MaAsLin2 analysis was performed to further correct for potential biological and technical confounders with uninfected animals as reference. *P* values were corrected for multiple testing using the Benjamini–Hochberg method and only species with a false discovery rate (FDR) < 5% were considered significant.
